# Boosting multifunctionality through adaptive trait‐based species addition in ongoing restoration projects

**DOI:** 10.1002/eap.70197

**Published:** 2026-02-24

**Authors:** André G. Coutinho, Alice Nunes, Cristina Branquinho, Vanderlei J. Debastiani, Marcos B. Carlucci, Marcus V. Cianciaruso

**Affiliations:** ^1^ Departamento de Ecologia, Instituto de Ciências Biológicas Universidade Federal de Goiás Goiânia Goiás Brazil; ^2^ Programa de Pós‐Graduação em Ecologia e Conservação Universidade Federal do Paraná Curitiba Paraná Brazil; ^3^ cE3c ‐ Center for Ecology, Evolution and Environmental Changes & CHANGE ‐ Global Change and Sustainability Institute, Faculdade de Ciências Universidade de Lisboa Campo Grande Lisbon Portugal; ^4^ Laboratório de Ecologia Funcional de Comunidades (LABEF), Departamento de Botânica Universidade Federal do Paraná Curitiba Paraná Brazil

**Keywords:** adaptive management, functional ecology, long‐term restoration, ongoing restoration, restoration monitoring, species selection, trait‐based restoration

## Abstract

As trait‐based restoration practices continue to gain momentum, there is still an absence of effective methods to monitor ongoing restoration and, if necessary, amend species composition to achieve multiple restoration targets. This challenge is even greater in long‐term restoration projects, as a result of different techniques and restoration strategies, leading to a heterogeneous landscape with different levels of ecosystem functions (multifunctionality). During the restoration process, it may be necessary to increase multifunctionality, or a particular ecosystem function, either from scratch or beyond what has already been provided by planted species or species established through natural regeneration. However, these aspects remain underexplored in restoration ecology, primarily because of the lack of operational frameworks. Using data from a 40‐year ongoing quarry restoration in Portugal, we evaluated current levels of multifunctionality and how to restore or increase drought resistance, fire resilience, pollination, seed dispersal, and vegetation structure. We found that multifunctionality varies significantly across restoration sites within the landscape. Natural regeneration plays a central role in maintaining current levels of multifunctionality, but we demonstrate that it can be considerably increased by trait‐based planting of additional individuals—whether of resident or new species—into restored sites. Furthermore, we show that enhanced levels of multifunctionality can be achieved in future restoration sites by using optimized species combinations. Our study provides important insights into the adaptive management of trait‐based restoration and provides a framework to achieve multiple objectives in ongoing restoration projects. We expect the proposed framework will enhance both the appeal and practical application of trait‐based and functional enrichment approaches in restoration practice.

## INTRODUCTION

The success of ecological restoration relies, in part, on establishing targets as references to determine whether restoration goals have been accomplished or not (Prach et al., 2019). Restoration projects may result in unsatisfactory outcomes if the restored community transitions to alternative states (Suding et al., 2004) or if the recovery is too slow (Nerlekar & Veldman, 2020; Shoo et al., 2016). Examples include the high mortality of planted individuals, the dominance of few species, or the emergence of invasive species (Reis et al., 2024). Thus, regular monitoring and post‐restoration interventions are often necessary (Camarretta et al., 2020). These include managing disturbances (Jones et al., 2018), eradicating invasive species (Weidlich et al., 2020), and/or adding missing native species (Mangueira et al., 2019). Understanding the appropriate intervention methods and their timing remains crucial in any restoration initiative. Species additions can be implemented at the outset of restoration, during ongoing restoration efforts, or in both stages (Arroyo‐Esquivel et al., 2023). However, evaluating the timing for suitable species introductions in ongoing restorations and the contribution of natural regeneration (Chazdon & Guariguata, 2016) to restoration objectives poses notable challenges in the field of restoration ecology. This is particularly true in the context of restoring ecosystem functions, where operational frameworks are relatively recent and still require further development to achieve the specificity and predictability needed to be useful for restoration practitioners (Merchant et al., 2023).

Determining the need for interventions in ongoing restorations, including suitable species for planting and the optimal timing for their introduction, relies on various factors. The first factor relates to the specific restoration objectives and their corresponding targets (Carlucci et al., 2020; Fremout et al., 2022). In this sense, restoring multiple ecosystem functions (hereafter, multifunctionality) has become an important goal in many restoration efforts (Cruz‐Alonso et al., 2019; Rosenfield & Müller, 2019), as biodiversity loss and habitat degradation are impairing the provision of ecosystem services worldwide (Cardinale et al., 2012; IPBES, 2018). Because recovering ecosystem functioning requires planting or adding species with adequate trait values (Laughlin, 2014), it becomes imperative to identify suitable species combinations to restore the desired functions (Coutinho et al., 2023). The species selection relies on a defined target, which may involve maximizing a functional aspect of the community (Laughlin, 2014), such as functional composition or functional diversity (Laughlin et al., 2018). Reference sites can also be used to define the adequate range of functional diversity or functional composition (Rosenfield & Müller, 2017). Nevertheless, even when the objective is to maximize functional aspects, reference sites should be considered to avoid creating novel ecosystems (Coutinho et al., 2023).

Landscape‐scale restoration can be planned in two ways: (i) by assuring that all restoration objectives are met at each site, thus creating “super‐communities” (Cianciaruso et al., 2025) in which all target functions are potentially restored; or (ii) by establishing complementary communities with different functions across restoration sites, thereby promoting spatial heterogeneity in ecosystem functions. When multiple functions are targeted, it may be difficult, or even impossible, to meet all objectives simultaneously, either because key species traits are negatively correlated (trade‐offs) or because they are unrelated, making it hard to identify species combinations capable of supporting all desired functions (Coutinho et al., 2023; Dias et al., 2013). Thus, the selection of species for planting should consider the aimed spatial heterogeneity within a restoration landscape (Fremout et al., 2022). For example, vegetation vulnerability to fire may vary spatially (Meira‐Neto et al., 2011), and in areas where fire is more likely to occur, fire‐resilient communities should be prioritized. Similarly, riverbank and floodplain restorations should emphasize the selection of species with traits that enhance soil retention, water, and nutrient cycling (Tabacchi et al., 2000). Therefore, successful restoration at the landscape scale depends on fulfilling different objectives in different locations.

Environmental heterogeneity in restoration landscapes can result in different species compositions in each restoration site because of different initial conditions and different temporal dynamics (Coutinho et al., 2019). Restoring diverse functions within these sites involves the use of specific sets of species (Laughlin, 2014), a consideration that is further influenced by the resident species composition, particularly in the context of intervening in an ongoing restoration effort (Rosenfield & Müller, 2019). In this case, the addition of new individuals, whether of resident or new species, must consider the composition and functional roles of already established species, as well as the surrounding natural vegetation, which may serve as a source for natural dispersal. However, incorporating these into restoration planning is not a trivial task, particularly when different sites are expected to prioritize different ecosystem functions. Ongoing restoration communities may consist of a combination of remaining, planted, and naturally regenerated species, which could result in trait values that do not significantly contribute to the targeted ecosystem functioning. For example, naturally regenerated species may accelerate the restoration if they have ecological traits that contribute to the desired functional targets (Wijedasa et al., 2020), but in other cases, they may have no substantial contribution to multifunctionality or even reduce it (Moi et al., 2021). The potential to enhance multifunctionality in landscapes under restoration, or already restored, through the establishment of new restoration sites may guide species selection, with functions that are currently underrepresented being prioritized in these new sites. Integrating all these aspects is challenging, and attempts to evaluate the contribution of planted species, naturally regenerated species, and those planned for addition to restoration sites to multifunctionality in ongoing restorations are scarce (but see Carvalho et al., 2022; Rosenfield & Müller, 2017).

Here, we employed for the first time a quantitative trait‐based approach to assess: (1) the success of an ongoing restoration in achieving multiple ecosystem functions, as well as to determine the extent and nature of the functions currently restored by planted and naturally regenerated species; (2) the potential for enhancing the multifunctionality in the landscape through the addition of new individuals, which may belong to either resident or new species, an aspect of ecological restoration that remains largely unexplored. As a case study, we show how multifunctionality can be quantified and increased in an ongoing Mediterranean shrubland restoration in a limestone quarry in Portugal, where restoration actions have been taking place since 1983. This long‐term restoration program applied different strategies over time, which created a heterogeneous landscape where each site has a distinct species composition, including a combination of planted and naturally regenerated species. We evaluate whether and how this heterogeneity determines current levels of multifunctionality considering fire resilience, drought resistance, vegetation structure, resources for pollinators, and seed dispersal potential across restoration sites. These aspects are aligned with the EU Biodiversity Strategy for 2030 (EC, 2021) and are important for the ecological restoration of Mediterranean shrublands that originally occurred in this quarry area because they are related to ecosystem resistance and resilience to disturbances (fire and drought), ecosystem functions (pollination and seed dispersal), and should also improve fauna conservation. More specifically, we answered the following questions: (1) Does the quarry undergoing restoration have levels of multifunctionality similar to those in reference sites? (2) What is the contribution of planted and naturally regenerated species to current levels of multifunctionality? (3) Could the introduction of new individuals and species to the current restoration sites enhance multifunctionality? and (4) What levels of multifunctionality can be achieved by implementing a trait‐based species selection framework in sites to be restored in the future? We used an upgraded version of the framework proposed by Coutinho et al. (2023) to find species that could increase multifunctionality in the restoration landscape. To demonstrate how to achieve specific targets when restoration objectives are explicitly spatialized, we prioritized fire resilience in sites located at the quarry boundaries that are more prone to burning.

## METHODS

### Study area

The restoration landscape is a limestone quarry area located in Serra da Arrábida, Portugal (between 38°27′ and 38°30′ N and between 8°55′ to 9°02′ W), within the Arrábida Natural Park (Appendix S1: Figure S1). The region is characterized by a dry sub‐humid climate, and Mediterranean shrublands predominantly surround the quarry. In this vegetation type, many plant species are adapted to fire and drought, which are common in the region (Meira‐Neto et al., 2011). The restoration in the quarry was initiated in 1983 to recover native vegetation, mitigate soil erosion, and minimize the adverse visual impact of exposed mining surfaces (Oliveira et al., 2014). To achieve these goals, several shrub species, mostly native, were produced locally and planted. In a subsequent phase, hydroseeding with herbaceous species was employed, primarily to prevent soil erosion on quarry slopes (Oliveira et al., 2014). The restoration was performed at different moments and using different techniques from 1983 to the present. From 1983 to around 2000, revegetation was performed mainly in horizontal 20‐m‐wide platforms which resulted from mining activities, through woody species plantations, mostly native (ca.15 species, e.g., *Arbutus unedo*, *Ceratonia siliqua*, *Juniperus phoenicea*, *Myrtus communis*, *Olea europaea*, *Phillyrea latifolia*, *P. angustifolia*, *Pistacia lentiscus*, and fast‐growing species like *Pinus halepensis*, *Retama monosperma*, and *Spartium junceum*—see Meira‐Neto et al., 2011 and Nunes et al., 2014 for more details). Since around 2000, quarrying methods have changed, allowing the creation of slopes 20 m high, and more recently, 10 m high. Revegetation of these slopes was carried out mainly with hydroseeding technique, using mostly herbaceous species. The hydroseeding mixture was initially dominated by more generalist species (e.g., *Dactylis glomerata*, *Festuca arundinacea*, *Lolium perenne*, *Trifolium repens*), which were progressively complemented or replaced by native species (e.g., *Brachypodium phoenicoides, Bituminaria bituminosa*, *Ononis natrix*) as a result of different restoration trials (see Oliveira et al., 2012, 2013, 2014 for more details). In some quarry areas, both planting and hydroseeding techniques were used, resulting in a heterogeneous landscape with different species compositions within the quarry (Oliveira et al., 2014).

To evaluate landscape multifunctionality (LM), we used six plant traits related to important ecosystem functions in Mediterranean shrublands and calculated the functional composition of 59 restoration sites selected within the quarry, where the plant community was sampled in Spring 2021 (Table 1). We used a subset of the regional plant species pool that includes 172 species adapted to the limestone soil found in the restoration region (Flora‐On, 2023) and from which trait data were available, in the literature or plant trait databases (Appendix S1: Box S1), or could be extrapolated to the genus level (11 species; Appendix S1: Table S1). Among the species in our regional pool, 26 were introduced through planting efforts in the restoration sites, 90 are native species that regenerated naturally over time, and 56 are native species only found in reference sites that could potentially be employed in future restoration efforts.

**TABLE 1:** Plant traits used in this study, their corresponding ecosystem functions, the significance of each function, the metric used to quantify functional composition, and the threshold values from which we considered the functions restored.

Trait	Function	Significance	Functional composition	Threshold
Zoochory	Seed dispersal/Resource for fauna	Zoochoric plants play a crucial role as a food source for various animals, many of which act as seed dispersers (e.g., birds and mammals) (Rigacci et al., 2021)	CWM of zoochory*	76%
Resprouting	Increase community fire resistance and resilience	Fire is a common anthropic disturbance in the region (Meira‐Neto et al., 2011), and resprouting increases the survival of individual plants after fires	CWM of resprouters*	66%
Entomophily	Abundance of resources for pollinators	Pollinators can support the natural regeneration process of a native ecosystem and are also an important source for food web (Neuschulz et al., 2016)	CWM of entomophily*	48%
Flowering duration (months)	Time availability of resources for pollinators	Pollinators can support the natural regeneration process of a native ecosystem and are also an important source for food web (Neuschulz et al., 2016)	CWM of flowering duration	3.8 months
Leaf mass per area (LMA)	Drought resistance	Drought is a natural disturbance in the region and plants with high LMA should be more resistant to drought events (Poorter et al., 2009)	CWM of LMA	0.095 mg mm^−2^
Plant height	Vegetation structural complexity	A high variability in plant height reflects vegetation structural complexity that tends to be positively correlated to biodiversity, by increasing available niche space for other flora and fauna species (Müller et al., 2014)	CWV of plant height	46531.74 m^2^

*Note*: Thresholds represent the average value of each function as observed in reference sites. Asterisk indicates binary traits, for which the CWM is equal to the percentage of the individuals with a given trait in the community. Note that, to calculate the variance, the trait difference to the CWM is squared, which results in high values and square meters as unit.

Abbreviations: CWM, community‐weighted mean (Garnier et al., 2004); CWV, community‐weighted variance (Hulshof et al., 2013); LM, landscape multifunctionality.

### Species selection and multifunctionality estimates

#### Evaluating the contribution of planted and naturally regenerated species to current multifunctionality

**FIGURE 1:**
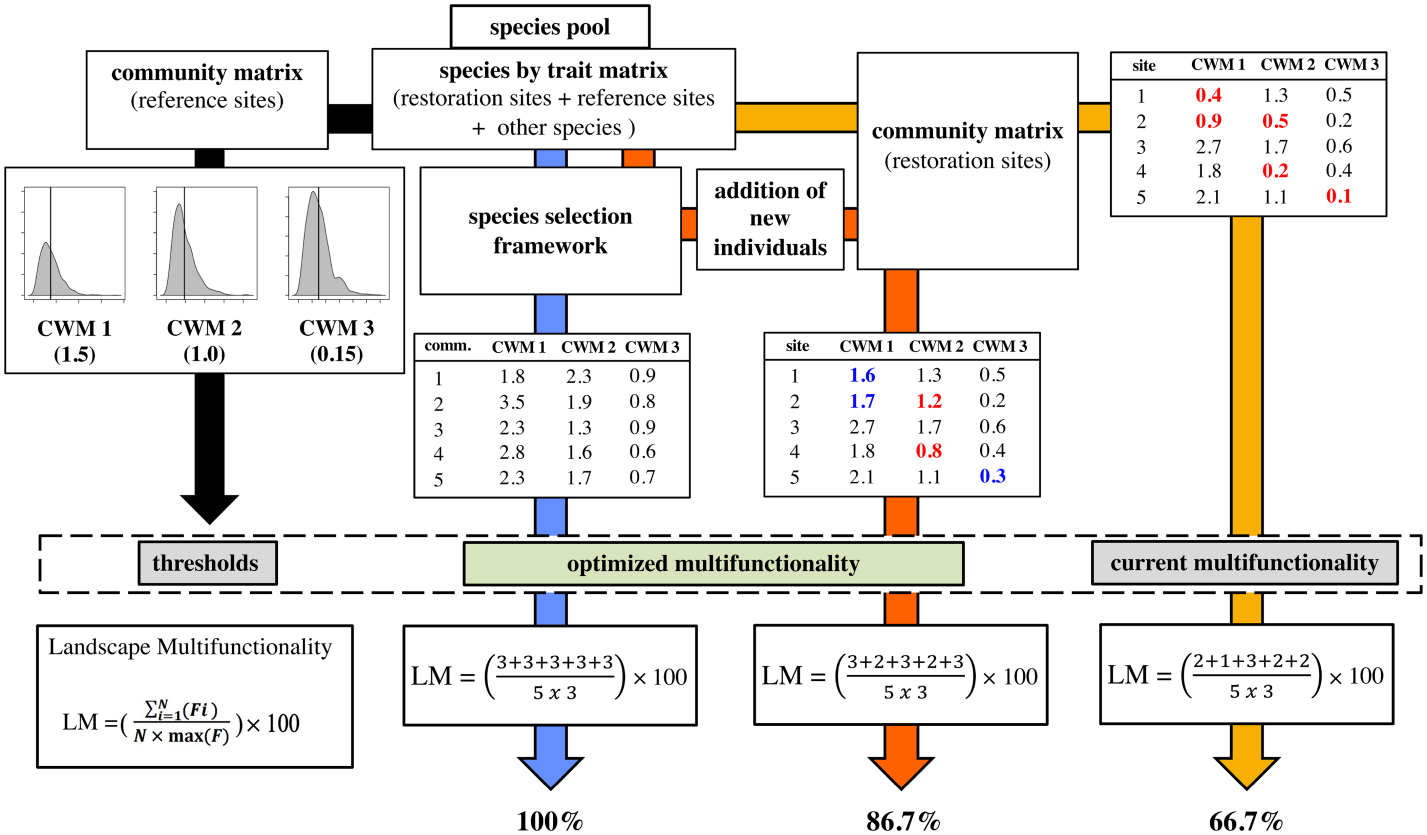
A conceptual scheme illustrates how the framework applied in this study operates. It begins with a species–trait matrix that includes all species in the species pool—specifically, species found in the restoration sites, species found in the reference sites, and any other species that are ecologically appropriate to incorporate—for example, species known to occur in the reference ecosystem within the region but not detected in the sampled reference sites. In this example, we simulate a scenario targeting three ecosystem functions for restoration, represented by their community‐weighted mean (CWM) trait values. Following the black arrow, we first calculate the CWM values for the reference sites and define restoration thresholds as the average CWM values across these sites (illustrated by the histograms on the left of the figure). A function is considered restored in a given site when its CWM value meets or exceeds the corresponding threshold. Following the yellow arrow, we evaluate current multifunctionality by calculating CWM values for restoration sites and counting how many functions exceed their respective thresholds. These results are shown in the matrix at the top right of the figure, where CWM values below the threshold are marked in red. Landscape multifunctionality is then computed using the formula shown, adapted from van der Plas et al. (2019). In this example, current multifunctionality is 66.7%. To enhance this value, the framework can be applied to simulate the addition of new individuals to the current restoration sites, while accounting for species abundances and co‐occurrence patterns (orange arrow). These additions may improve CWM values for some functions (e.g., CWM1 and CWM3 in blue), though not for others (those remaining in red). In this case, optimized multifunctionality increases to 86.7%. Finally, the blue arrow illustrates what could be achieved if the framework were applied from the outset of species selection for restoration. Again, controlling for observed species abundances and co‐occurrences, this scenario results in all functions exceeding their thresholds, thereby achieving maximum landscape multifunctionality (100%).

We updated a framework that selects species for restoration (Coutinho et al., 2023) to estimate and increase the number of ecosystem functions in ongoing restoration sites and, consequently, improve LM. The framework generates communities with distinct species compositions and abundances, which are randomly selected from a regional species pool (Figure 1). It requires species‐level trait data related to the ecosystem functions targeted in the restoration project for all species within the species pool. The species richness within simulated communities varies within a range defined by the user, and it is possible to calculate several functional aspects for each simulated community. These aspects include, for example, functional diversity and functional composition metrics (Coutinho et al., 2023). In the present study, our emphasis was on functional composition, using community‐weighted mean (CWM) and community‐weighted variance (CWV), calculated from different plant traits (Table 1), as proxies for ecosystem functions relevant to the restoration under evaluation (Garnier et al., 2004; Hulshof et al., 2013). We defined the multifunctionality of each restoration site as the number of functions above a given threshold (Table 1, Figure 1; Gamfeldt et al., 2008). The threshold indicates the level of functional composition above (or below) which the function is considered as restored in each site. For example, one may consider that a function is restored if the functional composition (e.g., CWM) is similar to that found in reference sites. This assumption is based on the premise that reference sites exhibit sufficient levels of ecosystem functioning (Rosenfield & Müller, 2019). However, our objective here was to achieve higher values of the desired functions in order to enhance ecosystem functions considered relevant to the restoration of Mediterranean shrublands. A threshold approach is adequate to calculate multifunctionality because it assumes that a decrease in one function cannot be compensated by an increase in another function (Gamfeldt et al., 2008). Here, we used data from 19 sites of Mediterranean shrublands located in the surroundings of the quarry (Arrábida Natural Park), where plant community composition was sampled in Spring 2021, as reference sites. We considered a given function to be recovered in the restoration sites when its corresponding CWM (or CWV) reached or exceeded the average level observed in reference sites (Table 1, Figure 1, Appendix S1: Figure S2). While some studies, such as van der Plas et al. (2019), define high functionality using a stricter benchmark (e.g., 90% of the 97.5th percentile), we opted to use the mean as an operationally attainable threshold. This choice aligns with our goal of optimizing function recovery while providing a clear and applicable framework for guiding species selection. Our framework, however, is flexible and can accommodate any desired threshold. As a rule of thumb, the stricter the threshold, the more challenging it is to recover multiple functions at a given restoration site. To assess LM, we calculated it across all 59 restoration sites within the quarry as follows:

(1) $$\mathrm{LM}=\left(\;\frac{\sum_{i=1}^{N}\left(\mathrm{Fi}\right)}{N\times F\max}\;\right)\times 100,$$

where *N* is the total number of sites, *F*_*i*_ is the number of functions at or above the thresholds in site *i* (which ranged from 0 to 6; see Table 1), and *F*max is the maximum number of functions. Thus, LM was calculated for the entire restoration area and is expressed as the amount of multifunctionality observed in relation to the maximum multifunctionality possible for the landscape (i.e., if all 59 restoration sites had all six functions restored).

To evaluate current levels of multifunctionality in the restoration landscape (Figure 1), we calculated LM using all species found in each restoration site (both planted and naturally regenerated), and then recalculated it excluding naturally regenerated species in order to assess their contribution to current LM. We also calculated LM for the set of 19 reference sites in order to have an estimate of the natural level of multifunctionality. Finally, we evaluated potential relationships between functions by examining their correlations, as well as the correlations among species traits. Additionally, we performed a principal coordinate analysis (PCoA) to order all species in the species pool based on their traits.

#### Increasing multifunctionality with the addition of individuals

To increase the multifunctionality in the restoration landscape, we updated the framework by Coutinho et al. (2023) to find species whose individuals could be added to increase the number of sites that restore different functions (Table 1, Figure 1). This step is based on generating random species compositions drawn from the regional species pool and requires: (1) a species–trait matrix for the regional species pool, (2) a community matrix with species abundance in reference sites, and (3) a community matrix with species abundance in ongoing restoration sites (Figure 1). The framework returns a community matrix indicating the proportion of individuals to be added for each species. The framework allows the addition of new individuals from any species within the species pool, including both resident species (already present at the site) and new species (not yet established). Following the simulated addition, the relative abundances of all species are recalculated. To avoid recommending high abundances of naturally rare species, sampling probabilities for each species are weighted by empirical data, in this case, the average relative abundances observed in reference and restoration sites. We note that absolute species abundances cannot decline in this simulation framework. A species‐level comparison between simulated and observed abundances is provided in Appendix S2. Furthermore, to prevent unrealistic combinations of species that do not naturally co‐occur, we excluded pairs of species whose co‐occurrences were significantly lower than expected by chance. Co‐occurrence probabilities were calculated using the *cooccur* function from the *cooccur* package in R, which implements the probabilistic model proposed by Veech (2013). These two constraints—controlling for species' typical abundances and observed co‐occurrence patterns—add ecological realism to the framework and, in principle, should improve the likelihood of success of the proposed planting mixtures.

To explore the relationship between the number of individuals added and the resulting LM, we ran the framework using varying levels of individual addition relative to the current plant density at each restoration site. These levels ranged from 10% to 200%, in 10% increment intervals, resulting in 20 rounds of simulations, each consisting of 5000 simulated communities. It is important to note that such levels of addition may be impractical in real‐world applications, as they could exceed the ecosystem's carrying capacity. Nonetheless, our objective was to assess the sensitivity of ecosystem functions to changes in community composition. Such an analysis can inform restoration practitioners about the potential multifunctionality gains associated with increasing planting density, while leaving it to their judgment to decide whether such an increase is feasible or whether initiating a new restoration effort from scratch would be more appropriate. Species richness was set between 10 and 26 species to correspond to the range observed in the reference sites. Despite being planted in certain restoration sites, *Pinus* species were not included in our simulations because they are non‐native and are no longer used by stakeholders in the studied restoration landscape. For each simulated community, we calculated how much of each function (Table 1) was restored. This update represents an enhancement to the original framework of trait‐based species selection for restoration (Coutinho et al., 2023) because it enables the incorporation of plant individuals from either established or new species into ongoing restoration sites.

To calculate the multifunctionality of each restoration site with new individuals added (Figure 1), we employed the same criteria used to evaluate the current multifunctionality. However, here we selected among the simulated communities those (if any) that increased the multifunctionality, so that one simulated winner community was chosen for each restoration site. For 51 out of the 59 ongoing restoration sites, we used higher multifunctionality as the criteria, that is, the framework returned a solution with the highest number of functions at or above their respective thresholds for each one of these restoration sites. The remaining eight sites were partially burnt in 2014 and are located near the quarry boundaries, making them more susceptible to wildfires (Appendix S1: Figure S1). Because of this, in these sites, we prioritized fire resilience over the other ecosystem functions by using fire resilience and multifunctionality as the criteria, in this order. Using the results of all simulation rounds, we compared the level of LM and landscape fire resilience with the number of individuals added, in order to identify the minimum required to reach the levels of reference sites. We also evaluated how many new species would be necessary to achieve each of these levels.

#### Selecting species compositions to optimize multifunctionality in future restoration sites

To recommend species compositions for restoring new sites from scratch, we used the original version of the framework (Coutinho et al., 2023), enhanced with constraints to control for species' typical abundances and observed co‐occurrence patterns (as previously explained), to identify simulated communities that optimize multifunctionality. We created 20,000 simulated communities, with species richness ranging from 10 to 26. Then, we selected the simulated communities with maximum multifunctionality and investigated how many of them contained species already planted in the restoration landscape and how many contained at least one species that was not planted. We also illustrate all the potential function combinations identified. The results of this simulation show how multifunctionality could be increased if the framework was used since the beginning of the restoration or in the restoration of new sites in the future. The results also indicate which species should be incorporated into local nurseries or collected from the wild (e.g., as seeds) to improve future restoration efforts.

To visualize multifunctionality, we used the *upset* function from the ComplexUpset R package (Krassowski, 2020), which creates a graphical representation of the number of sites where each function has been restored (i.e., it is at or above the threshold), and the number of sites where distinct combinations of functions have been restored. The updated framework was built in R 4.4.2 (R Core Team). The script and the data used to run the analyses are available in Coutinho et al. (2025).

## RESULTS

**FIGURE 2:**
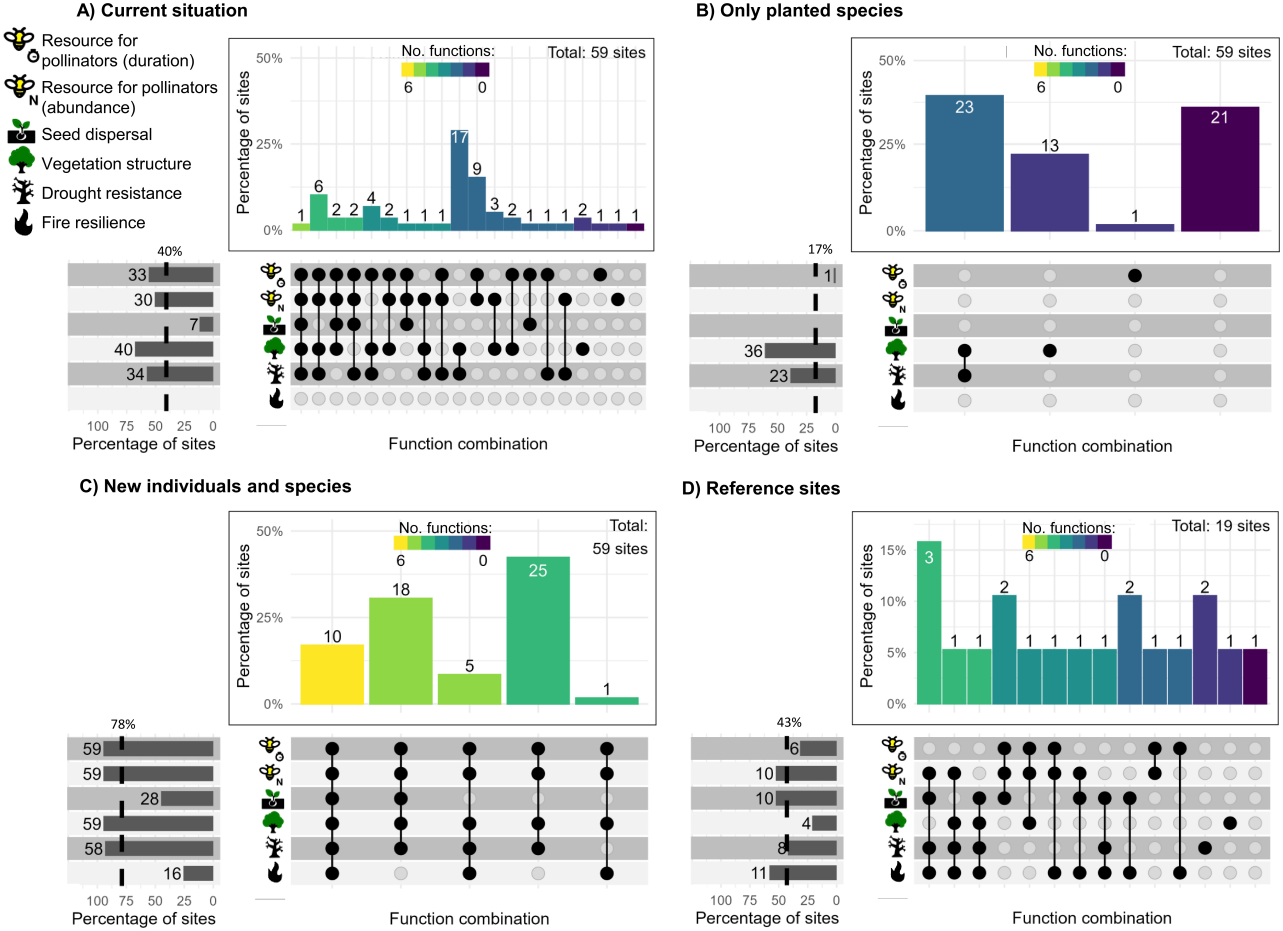
Multifunctionality in four different scenarios in a quarry restoration area. Horizontal bars show the percentage of sites surpassing the predefined thresholds, with the absolute number of sites indicated by the number on the left. Dashed lines represent the average percentage number of sites where the functions are restored, which is simply the average of the horizontal bars, and represent the level of landscape multifunctionality. Vertical bars show the percentage of sites where combinations of functions (dots below the vertical bars) are above the thresholds. Panel (A) shows the current situation in the entire restoration area. Panel (B) shows the contribution of planted species to multifunctionality after excluding naturally regenerated species. Panel (C) shows the multifunctionality with the addition of 120% of new individuals according to the proposed framework, which is the minimum amount of new individuals necessary to restore fire resilience in border sites. Panel (D) shows the multifunctionality of the reference sites. Note that because the thresholds are defined using the average level of the functions in reference sites, some of them may be below this threshold. Numbers above and on the left of the bars represent the absolute number of sites where the function, or a combination of functions, has been restored. Icons in the figure were created by André Coutinho.

In the current state of landscape restoration, without any quantitative trait‐based intervention to reach specific ecological goals, ecosystem functions have been restored to 40.7% of the maximum potential LM (Figure 2A). We found considerable variability among sites concerning restored functions, with the number varying from zero to five at each site (out of a maximum of six functions). Fire resilience is not restored at any site, while vegetation structure is successfully restored in 68% of the sites (Figure 2A). When naturally regenerated species were excluded, multifunctionality decreased from 40.7% to 16.9%, with the maximum number of functions restored in a single site reduced to two (Figure 2B). Without naturally regenerated species, more than a third of the restoration sites would not have restored any ecosystem function, whereas drought resistance and vegetation structure would be restored only to a certain degree (Figure 2B). The effect of naturally regenerated species on multifunctionality is disproportionate to their abundance, as planted and naturally regenerated species have, on average, nearly the same number of individuals in the restoration sites (Appendix S1: Figure S3). However, even though the current level of multifunctionality (40.7%, Figure 2A) is just below that observed in reference sites (43%), it is noteworthy that fire resilience—an ecosystem function present in over half of the reference sites (Figure 2D)—is yet to be recovered in the restoration sites.

**FIGURE 3:**
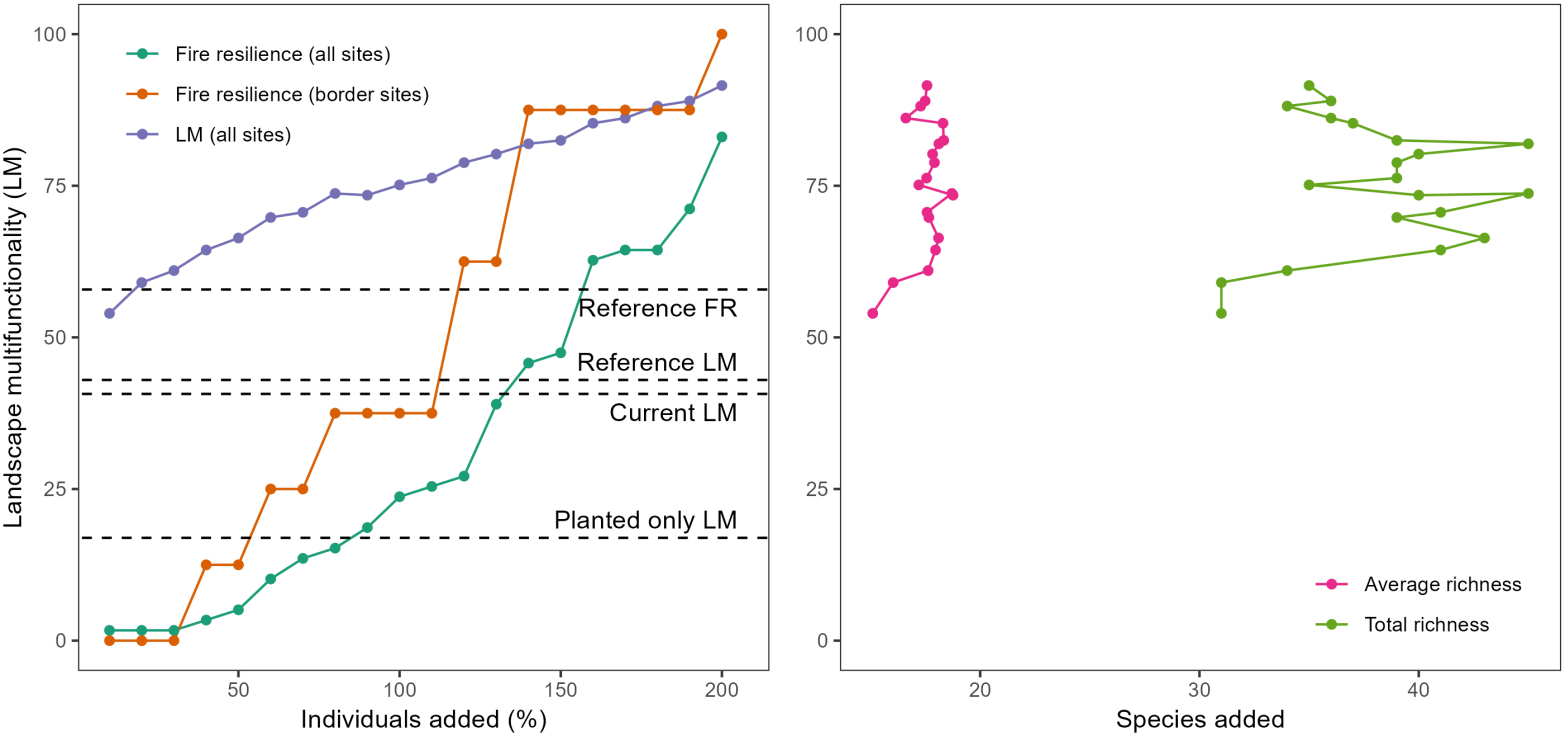
Effect of the percentage of individuals added (A) and new species added (B) on landscape multifunctionality (LM) in a quarry restoration area, based on 20 rounds of simulation using the proposed framework, with each round representing a different number of individuals added. Species richness is calculated as the average of new species added to the restoration sites (pink curve) and the total number added to the restoration landscape (green curve), according to the simulation output. The framework algorithm identifies which species, from the entire species pool, should receive additional individuals to increase LM or a specific function, such as fire resilience (FR). Dashed lines represent reference thresholds for LM and FR, current LM in restored sites, and LM considering only planted species. See the main text for details.

When we simulated the addition of individuals to ongoing restoration sites (Figure 2C), LM rapidly surpassed the levels of reference sites. With the addition of just 10% of the current individuals in the restoration sites, multifunctionality rose to 54% (Figure 3A). We observed a linear increase in multifunctionality as the number of individuals added to the restoration sites increased. This pattern suggests that higher densities lead to progressively higher multifunctionality values, with a projected maximum multifunctionality of 91.5% if the number of current individuals were tripled (200%, Figure 3A). Restoring fire resilience in border sites (which were burnt in 2014) to levels above the reference sites was only possible with the addition of at least 120% new individuals (Figures 2C and 3A), with an average of nine species per site that were not originally planted and a total of 33 new species at the landscape level (Figure 3B). In this case, multifunctionality increased to 79%, with fire resilience being restored in 62.5% of the border sites (Figure 3A), and ecosystem functions ranging from four to six per site (Figure 2C). This would also lead to 10 “super‐communities” with all six ecosystem functions restored (Figure 2C). These values are significantly higher than the reference sites, where only 26.3% of the sites have four functions at or above the threshold, with none having more than four functions (Figure 2D). However, even with this level of individual addition to ongoing restorations (120%), fire resilience would be restored in only 27.1% of the sites in the entire landscape (Figures 2C and 3A). Achieving fire resilience across all border sites would require tripling the current number of individuals in the ongoing restoration sites (Figure 3A). With this substantial increase, it would enable 83% of the restoration sites within the landscape to restore this ecosystem function, surpassing the level found in reference sites (Figure 3A). We found no strong relationships among ecosystem functions in the restoration sites, nor among species traits in the species pool (Appendix S1: Figures S4 and S5). However, the PCoA (Appendix S1: Figure S4) shows that resprouter species occupy only part of the trait space and are absent from regions associated with long flowering duration and/or zoochory.

**FIGURE 4:**
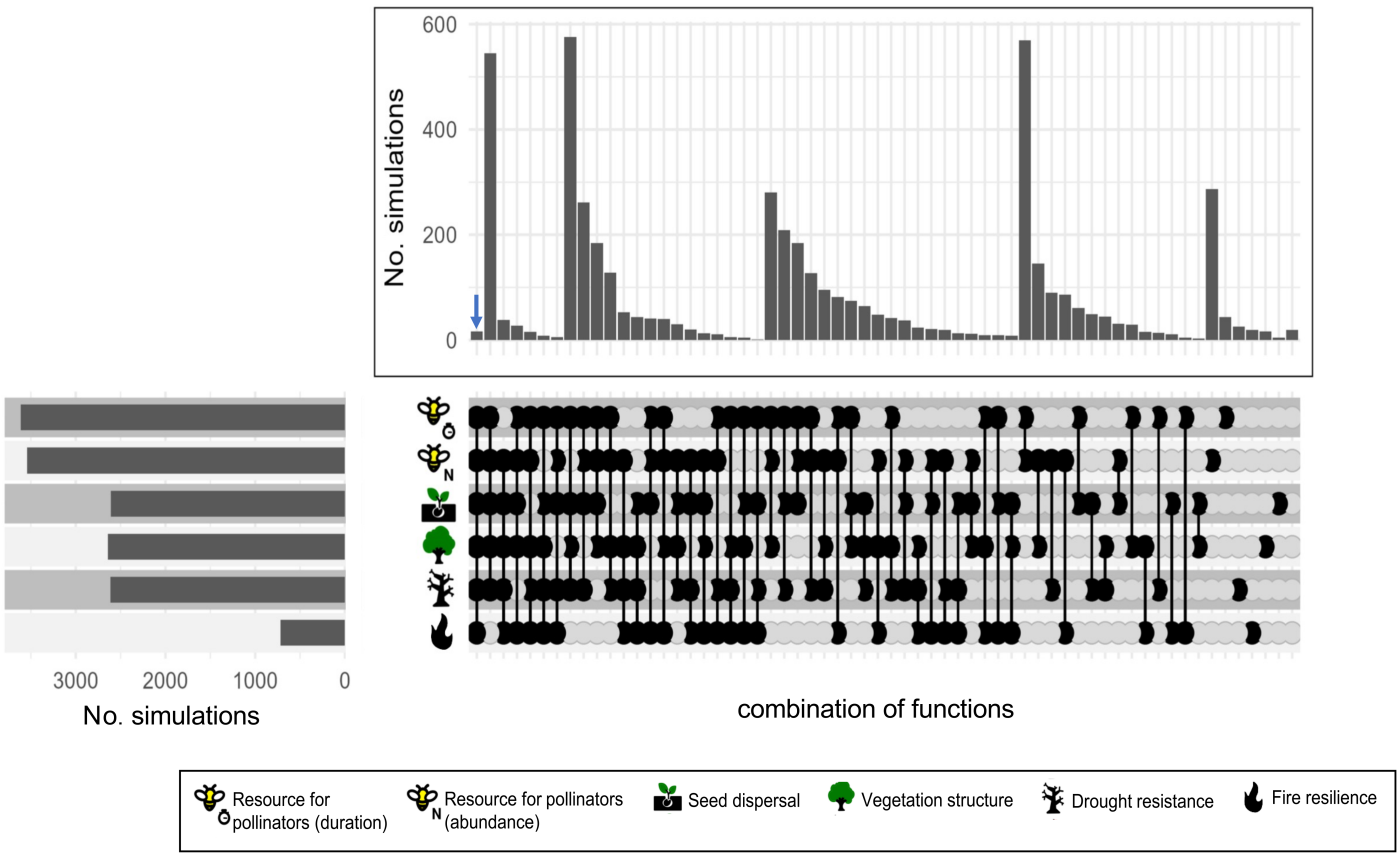
Multifunctionality in 5000 simulated communities, representing potential outcomes had the framework been applied at the start of the restoration project—or if used for future restoration sites. The blue arrow indicates the 17 simulated communities capable of restoring all six functions (“supercommunities”). A wide range of communities with distinct functional combinations could be implemented across the landscape; however, fire resilience remains the most restrictive function to recover. Icons in the figure were created by André Coutinho.

Finally, we simulated potential outcomes in a scenario where the framework had been applied at the start of the restoration project (or in future restoration sites) and identified 17 “super‐communities” capable of restoring all targeted ecosystem functions (Figure 4). Among these, 12 consisted solely of species originally planted in the restoration sites, while the remaining five have at least one species not originally planted, resulting in 30 species not used in the original restoration project. These 17 “super‐communities” have between seven and 17 species each, encompassing a total of 52 species (30% of the species pool used in the analyses). Notably, fire resilience was restored in significantly fewer simulations than the other functions (Figure 4). Beyond identifying communities that restore all functions, the simulated communities offer flexible options to enhance LM through diverse species mixtures that supply different combinations of functions. These alternative communities not only provide opportunities to expand the number of species used in restoration but also allow for functionally complementary assemblages that, together, can boost overall multifunctionality even if each restores fewer than six functions individually (Figure 4).

## DISCUSSION

We demonstrated how restoration ecologists and practitioners can assess the contributions of planted and naturally regenerated species to the multifunctionality of ongoing restoration projects. We illustrated how to identify plant individuals that enhance multifunctionality when added to existing sites or optimize it from the outset of future projects. Additionally, we showed how different restoration sites within a landscape can prioritize distinct ecosystem functions. Our findings indicate that seed dispersal, resources for pollinators, vegetation structure, fire resilience, and drought resistance vary significantly among the study restoration sites and are largely maintained by naturally regenerated species, but some functions are more difficult to recover than others. Despite naturally regenerated species maintaining multifunctionality levels similar to those of reference sites, we demonstrated that these levels could be enhanced by planting individuals of new species or increasing the number of individuals of those already established in the restoration sites. Moreover, restoring new sites in the quarry area can be achieved through species combinations that restore all functions (“super‐communities”). Our study highlights important aspects that should be considered when restoring and monitoring multifunctionality in ongoing and future ecological restorations, and demonstrates ways to address them.

Evaluating and monitoring the contribution of naturally regenerated species to the restoration outcome are important because their impact can be unpredictable, given that species dispersal and arrival can be stochastic (Fukami, 2015), having positive (Prach et al., 2014) or negative effects (Questad et al., 2012). In our study area, natural regeneration had positive effects, contributing to more than half of the current multifunctionality and accounting for 78% of the species found in the restoration landscape. Naturally regenerated species likely dispersed from the native vegetation of the Arrábida Natural Park that surrounds the limestone quarry under restoration. Such findings are clear evidence of the importance of natural regeneration to the recovery of ecosystem multifunctionality and the need to protect, conserve, and connect natural areas that can serve as a source of native species (Chazdon & Guariguata, 2016; Prach et al., 2014).

Restoring heavily degraded sites, such as quarry areas, involves significant challenges because of the complete removal of vegetation and the loss of soil resulting from rock extraction. In such cases, relying exclusively on natural regeneration may not be feasible within a reasonable time frame. Correia et al. (2001) found, within the same limestone quarry we studied here, that active restoration facilitates the rapid establishment of species compared to sites under passive restoration. In our study case, planting species was necessary to minimize soil erosion and create microclimatic conditions that facilitate the colonization of species arriving from natural areas (Correia et al., 2001). Nevertheless, our findings indicate that neither planted species nor natural regeneration was capable of restoring fire resilience to the levels observed in the reference ecosystem. Given that fire is a common anthropogenic disturbance in the region (Meira‐Neto et al., 2011) and is expected to increase due to climate change, restoration practitioners focusing on fire resilience in Mediterranean shrublands need to exercise caution when relying solely on natural regeneration, especially if increasing the number of resprouters is one of the restoration objectives.

Planted and naturally regenerated species together were capable of restoring the multifunctionality to levels similar to reference sites, on average, but differed a lot in terms of the functions restored. While the duration of resources for pollinators, vegetation structure, and drought resistance was substantially improved, seed dispersal, drought resistance, and fire resilience were considerably reduced. This result highlights the ongoing need for assisted restoration and continuous monitoring to further enhance LM and achieve specific ecosystem service targets. If the current levels of multifunctionality in ongoing restorations are below the defined thresholds, planting new individuals is a straightforward solution to guide the restoration back onto the desired trajectory (Cianciaruso et al., 2025; Rosenfield & Müller, 2017). We demonstrated that surpassing the multifunctionality of the reference ecosystem by a significant margin is achievable by adding just 10% or 20% more individuals to the ongoing restoration sites. Planting new individuals could potentially double the landscape's multifunctionality under the extreme scenario of adding 200% more individuals, although this is likely to be impractical in real‐world restoration efforts due to constraints such as the system's carrying capacity.

Restoring multifunctionality is challenging because, as the number of target functions increases, it becomes less likely that all of them can be recovered at a given restoration site (Gamfeldt et al., 2008; van der Plas et al., 2019). Considering the heterogeneity among restoration sites, as we did, makes it more feasible to restore all functions across the restoration landscape by restoring different functions in different sites (Schulz & Schröder, 2017). However, if a given function is significantly underrepresented in the restoration sites, it may be necessary to add so many new individuals that correcting the restoration trajectory over time or changing the original restoration objectives essentially becomes a planting effort similar to a new restoration initiative. This requires careful consideration by restoration practitioners and may lead to two decision paths. First, if a specific function must be restored across many sites in the landscape, it may be more effective to select species from scratch, creating new communities that prioritize the target function or “super‐communities” that encompass all functions. Alternatively, if the goal is to restore a particular function in specific sites, planting a relatively large number of individuals may be a worthwhile strategy. The latter was the case for fire resilience in the restoration landscape studied. Restoring fire resilience above the reference site's threshold would require planting 120% more individuals and introducing 33 species that were not originally planted in the restoration sites. However, achieving fire resilience in restoration sites located at the edges of the restoration landscape, where susceptibility to anthropogenic fires is higher, would require increasing individual abundance by up to 200%. Such an increase may be unfeasible, as it could exceed the ecosystem's carrying capacity or be constrained by other factors, such as competition with dominant species or limited soil resources. Furthermore, increasing plant abundance may lead to greater biomass accumulation, which could in turn elevate fire intensity, a trade‐off that must be carefully considered when aiming to enhance fire resilience.

Fire resilience was notably difficult to restore, especially in combination with the other targeted functions. This outcome can be attributed to the fact that, on average, restoration sites contain only half the abundance of resprouter species found in the reference ecosystem, making our threshold difficult to meet—particularly given that most species in the pool are non‐resprouters. This also raises the possibility of a trade‐off between resprouting ability and the other functions we investigated. Although no strong pairwise correlations or unidimensional trade‐offs were detected among traits, the multivariate trait space (PCoA) reveals a form of functional constraint: certain trait combinations—such as resprouting ability combined with long flowering duration and/or zoochory—are poorly represented in the species pool. This suggests that some functional strategies may be inherently limited or entirely absent, thereby constraining the potential to achieve multiple restoration objectives simultaneously. From a landscape restoration perspective, identifying such constraints is essential for determining where specific functions should be prioritized and whether functional enrichment is a viable strategy, or if initiating restoration from scratch would be more appropriate. Although restoring fire resilience proved challenging for the reasons outlined above, our results indicate that it would, in fact, be possible to achieve all targeted functions if species selection had been guided by our framework from the outset—even using only the originally planted species. These species span the entire functional trait space and include a broad range of trait combinations (Appendix S1: Figure S4). Therefore, the current inability of planted species to deliver high levels of multifunctionality in the restoration landscape is not due to a lack of functional traits, but rather to suboptimal species combinations and relative abundances, factors that can be improved by redesigning the species composition from the ground up. This would allow for greater multifunctionality from the beginning of the restoration initiative, even if, over time, these planted species are gradually replaced by naturally regenerating ones.

Taking into account a spatial dimension in restoration planning is useful for reconciling conflicting objectives and spatially targeted goals. For example, erosion control at the edges of roads may be necessary if they are present within restoration areas (Sandercock et al., 2017). Flower strips and hedgerows within agricultural landscapes can improve pollination services, pollinator diversity, and pest control (Dainese et al., 2017; Williams et al., 2015), while planting forest species with high wood density in other parts of the landscape may increase carbon stocks and help mitigate climate change at intermediary to long terms of restoration (Carlucci et al., 2020). At large scales, the optimal spatial distribution of ecosystem services depends on the demand and viability of restoring specific ecosystem functions. For example, Schulz and Schröder (2017) generated a map of multifunctionality hotspots in Central Chile, identifying areas with higher potential for restoring habitat function, erosion control, and carbon storage. The approach we employed here has an important advantage because it can be used to identify species compositions with combinations of traits that optimize distinct ecosystem services at both local and landscape scales. Restoring sites with complementary functions can be especially useful for large‐scale restoration because it is often performed at different moments, with different techniques, and involves different stakeholders that may have different restoration goals (Cruz‐Alonso et al., 2019; Hobbs et al., 2014). The same rationale applies to monitoring restored sites and improving or changing their targets over time. Indeed, the EU Biodiversity Strategy for 2030 (EC, 2021) primary goals include halting biodiversity loss, enhancing ecosystem resilience, and ensuring that Europe's biodiversity can sustainably provide essential ecosystem services. Among its main biodiversity targets are the conservation and restoration of habitats, with a special focus on pollination services and resilience against wildfires and droughts, which are likely to increase with climate change. These targets involve ecosystem functions explored in the present study and our framework could be used as a road map that allows restoration practitioners and ecologists to (i) evaluate which habitats and restored sites already achieve specific targets, (ii) select species that can be used to functionally enrich ongoing (and past) restoration sites in order to improve the likelihood they achieve the defined targets, (iii) inform new restoration efforts with multiple species compositions that would help to achieve the targets. Following these steps could be highly valuable for improving restoration success over time, as restored communities are expected to face environmental changes in the near future driven by anthropogenic activities (Cook‐Patton et al., 2021). We emphasize the importance of monitoring, as the temporal dynamics of planted species may change over time. While controlling for species abundances and coexistence enhances the realism of the solutions proposed by our framework, natural communities are inherently complex, and factors not included in our approach may influence outcomes as time progresses. For example, species' absolute abundances cannot decline in our simulation framework, as it only accounts for the addition of individuals. Although the framework can be applied during monitoring to track changes in parameter trajectories resulting from shifts in species abundances or composition over time, it can only address these changes by recommending the addition of new individuals. A promising avenue for future development would be to simulate restoration outcomes under scenarios in which particular species or individuals are removed. Nevertheless, our proposed road map provides an important step toward the adaptive management of trait‐based restoration, offering practical solutions for ongoing restoration efforts.

Recently, Merchant et al. (2023) raised the question of why traits are not often explicitly considered by restoration practitioners. Indeed, few restoration efforts explicitly adopt trait‐based approaches, particularly during the planning phase (Carlucci et al., 2020). This is partly because of the lack of specificity and predictability of existing frameworks (Merchant et al., 2023), which hinders their application in the inherently complex process of community assembly in restored ecosystems. Here, we advance on some of the issues highlighted in Merchant et al. (2023) by expanding the application of a recently published framework for species selection (Coutinho et al., 2023), addressing essential aspects to be considered during restoration and the monitoring of ecosystem functions. Our approach reconciles both active and passive restoration by offering a quantitative framework to assess the impact of planted and naturally regenerated species on ecosystem multifunctionality while using the reference ecosystem as a baseline. Additionally, it identifies whether, when, and where individuals should be added, either during the monitoring of ongoing restoration sites or as part of new communities for future restoration at both local and landscape scales. We anticipate that this approach, along with the insights presented here, will make trait‐based strategies more accessible to restoration ecologists and practitioners, thereby enhancing restoration efforts globally and contributing to the expansion of restoration scale and its positive impacts on biodiversity and ecosystem services delivery.

## AUTHOR CONTRIBUTIONS

All authors conceived the ideas, methods, and analyses. André G. Coutinho and Vanderlei J. Debastiani developed the R functions, with conceptual development of the framework by Marcus V. Cianciaruso and André G. Coutinho. André G. Coutinho wrote the first draft of the manuscript with input from all authors. All authors contributed to the final version of the manuscript.

## CONFLICT OF INTEREST STATEMENT

The authors declare no conflicts of interest.

## Supporting information


Appendix S1.

Appendix S2.

## Data Availability

Data and code (Coutinho et al., 2025) are available in Figshare at https://doi.org/10.6084/m9.figshare.27640566.
