# Supplementary material for: Boosting multifunctionality through adaptive trait‐based species addition in ongoing restoration projects
Source: Ecol Appl. 2026 Feb 24;36(1):e70197. doi: 10.1002/eap.70197 (PMC12931364; doi:10.1002/eap.70197)
Supplement: Supplementary file 1 — Appendix S1. [file EAP-36-e70197-s002.pdf]

Appendix S1

Ecological Applications

Boosting multifunctionality through adaptive trait-based species addition in ongoing restoration projects

André G. Coutinho, Alice Nunes, Cristina Branquinho, Vanderlei J. Debastiani, Marcos B. Carlucci, Marcus V. Cianciaruso

Box S1. List of trait source information used in this study.

Source
Kattge, J, Boenisch, G, Diaz, S, et al. TRY plant trait database - enhanced coverage and open access. Glob Change Biol. 2020; 26: 119-188. https://doi.org/10.1111/gcb.14904
Kleyer, M., Bekker, R. M., Knevel, I. C., Bakker, J. P., Thompson, K., Sonnenschein, M., ... & Peco, B. (2008). The LEDA Traitbase: a database of life-history traits of the Northwest European flora. Journal of ecology, 96(6), 1266-1274.
Maitner, B. S., Boyle, B., Casler, N., Condit, R., Donoghue, J., Durán, S. M., ... & Enquist, B. J. (2018). The bien r package: A tool to access the Botanical Information and Ecology Network (BIEN) database. Methods in Ecology and Evolution, 9(2), 373- 379.
Nunes A., Oliveira M.A.G., Mendes C., Oliveira G., Príncipe A., Serrano H., Clemente A., Serafim J., Cotrim H., Domingues I., Monteiro J., Kotzen B., Correia O. & C. Branquinho, 2021. Project Final Report: Adaptive management of ecosystems based on the evaluation of the resilience of more than 30 years of Ecological Restoration at Secil-Outão (2019-2021).
Tavşanoğlu, Ç., & Pausas, J. G. (2018). A functional trait database for Mediterranean Basin plants. Scientific Data, 5(1), 1-18.

Table S1. Percentage of trait data obtained at the species level and extrapolated to the genus level.

Trait	Level	
	Species	Genus
SLA	97	3
Height	98	2
Flowering duration	97	3
Entomophily	97	3
Zoochory	97	3
Resprouter	98	2

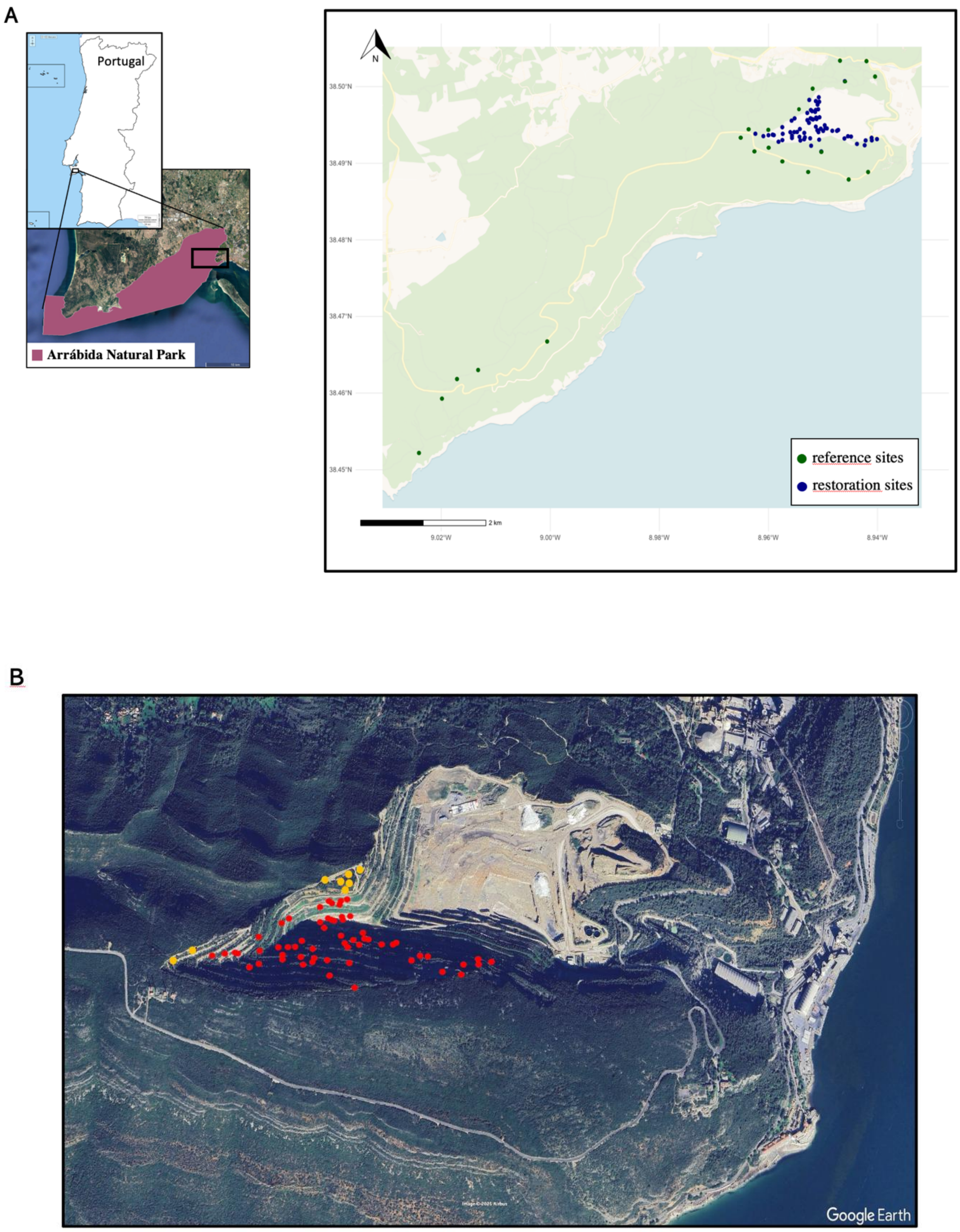

Figure S1. (A) Restoration landscape showing the restoration and reference sites located in Serra da Arrábida, Portugal (between 38°27′–38°30′ N and 8°55′–9°02′ W), within the Arrábida Natural Park. (B) Spatial distribution of restoration sites (red dots) and restoration sites along the border where fire resistance was prioritized for recovery (dark yellow dots) in the limestone quarry under restoration since 1983 (SECIL-Outão). Maps were generated using the maptiles package in R and Google Earth.

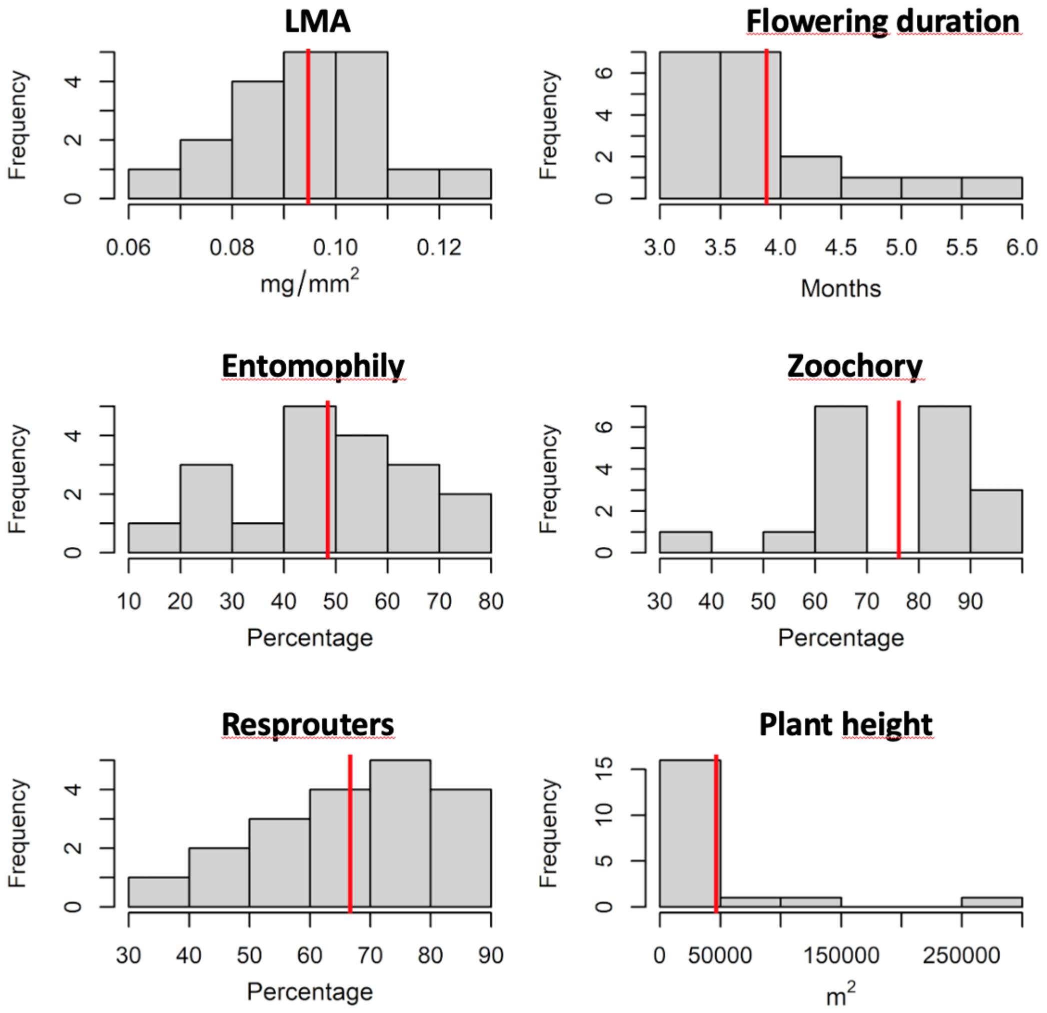

Figure S2. Trait distributions across 19 reference sites in a quarry restoration area in Arrábida National Park (Portugal). Solid red lines indicate mean values, which were used as threshold criteria in the analyses (see Table 1). All traits are expressed as community-weighted means (CWM), except for plant height, which is expressed as community-weighted variance (CWV).

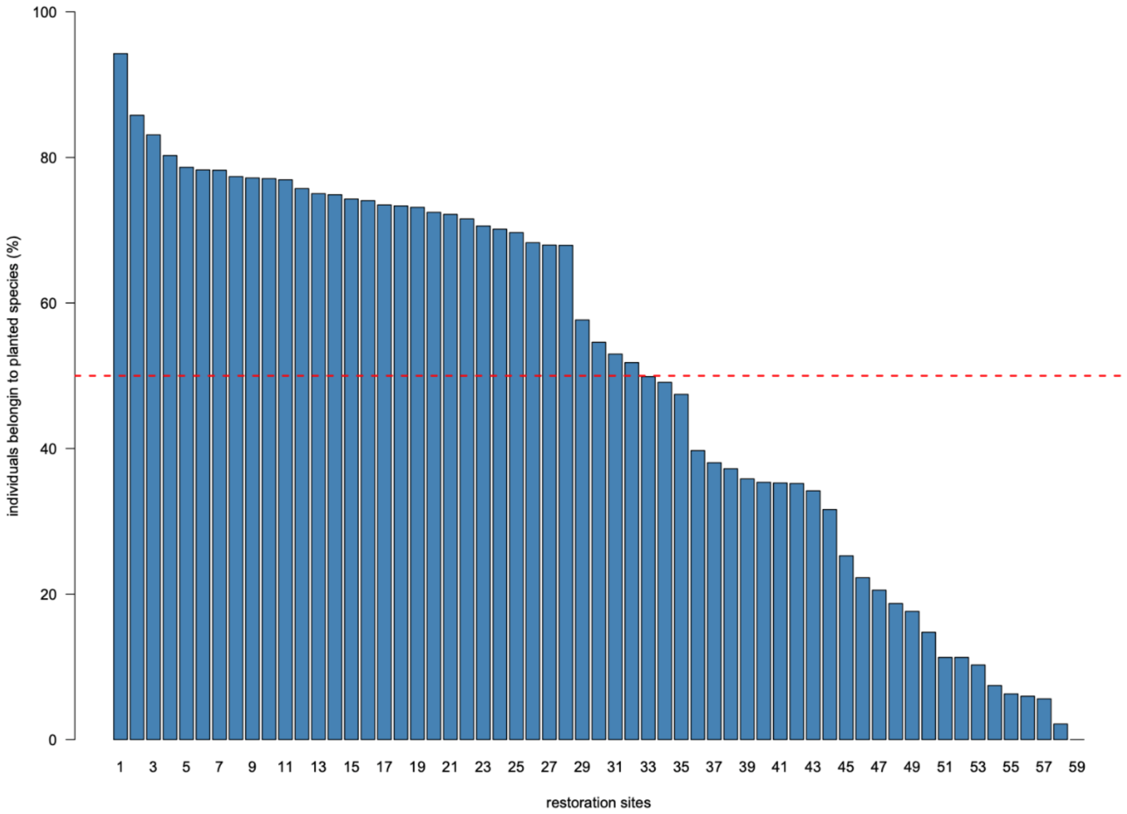

Figure S3. Proportion of individuals from planted species across 59 restoration sites, expressed as a percentage of the total number of individuals in each site. The red dashed line represents the average percentage (52%) of planted species individuals across all restoration sites.

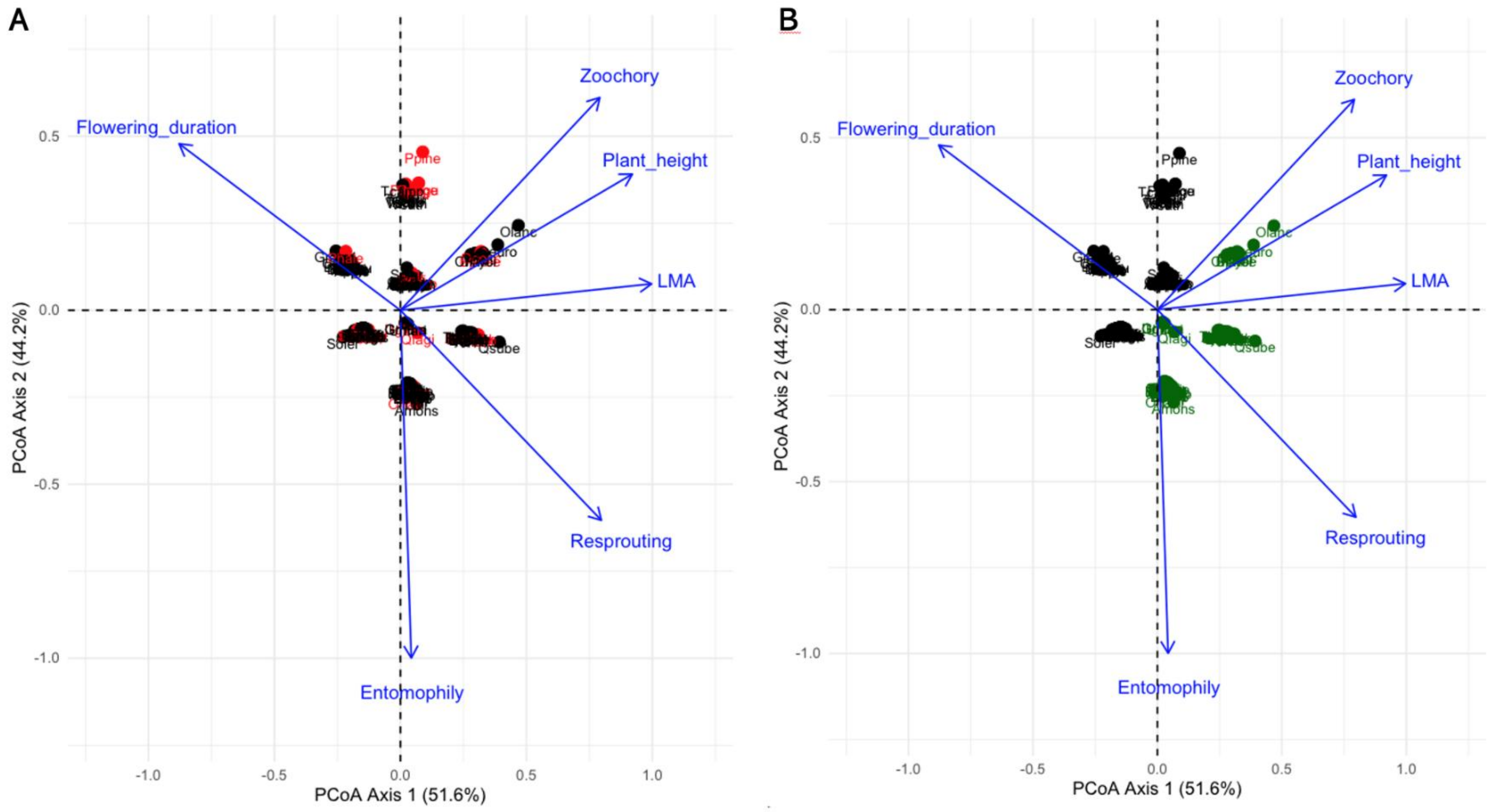

Figure S4. Species ordination (Gower distances) based on the six traits used in the analyses. (A) Species marked in red are those originally planted in the restoration sites. (B) Species marked in green are resprouter species, which comprise 36.6% of the species pool. While species originally used in restoration are distributed across the entire functional space (A), resprouters are restricted to specific regions of this space (B), particularly those associated with high leaf mass per area (LMA), greater plant height, zoochory, and entomophily. Notably, resprouter species are generally not associated with long flowering durations.

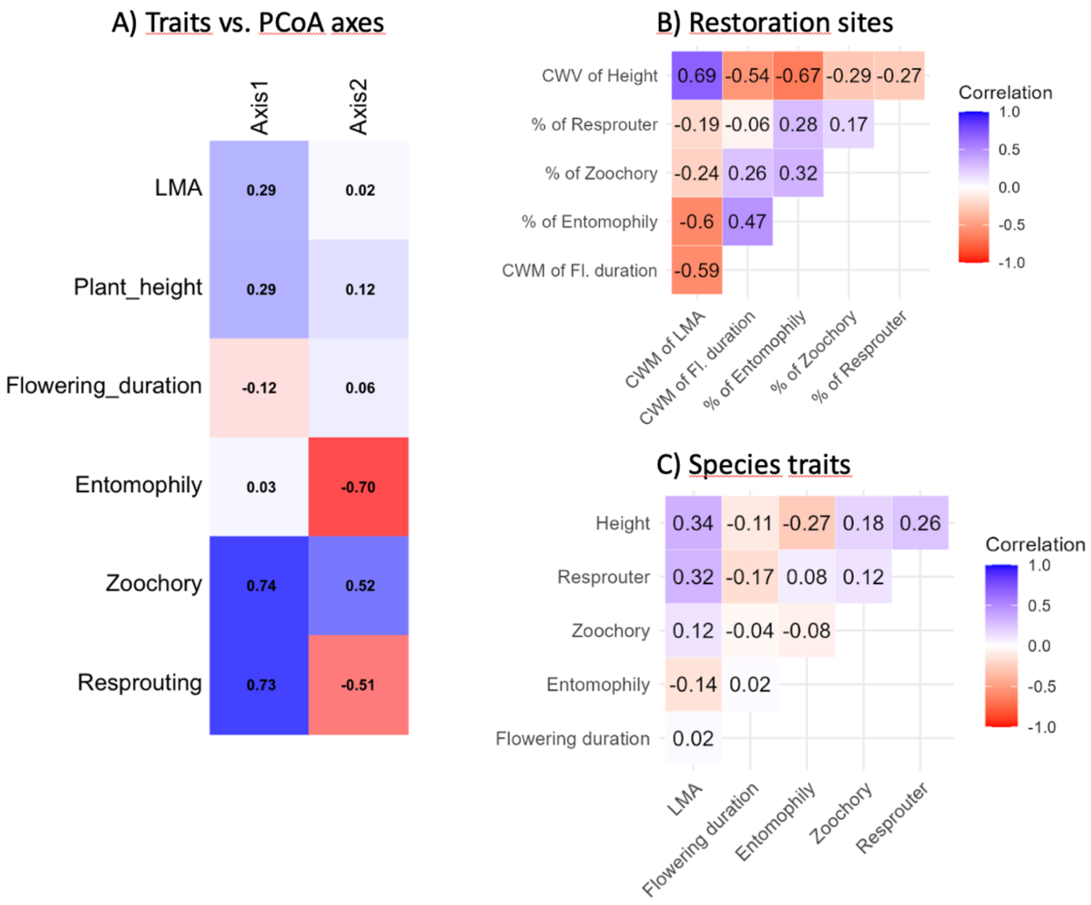

Figure S5. (A) Correlations between species traits and ordination axes (PCoA; see Figure S4); (B) correlations among community-level trait values across restoration sites; and (C) correlations among species-level traits. Pearson, point-biserial, and phi correlation coefficients were used for relationships between numeric–numeric, numeric–categorical, and categorical–categorical variables, respectively.
