# Supplementary material for: Boosting multifunctionality through adaptive trait‐based species addition in ongoing restoration projects
Source: Ecol Appl. 2026 Feb 24;36(1):e70197. doi: 10.1002/eap.70197 (PMC12931364; doi:10.1002/eap.70197)
Supplement: Supplementary file 2 — Appendix S2. [file EAP-36-e70197-s001.pdf]

Appendix S2

Ecological Applications

Boosting multifunctionality through adaptive trait-based species addition in ongoing restoration projects

André G. Coutinho, Alice Nunes, Cristina Branquinho, Vanderlei J. Debastiani, Marcos B. Carlucci, Marcus V. Cianciaruso

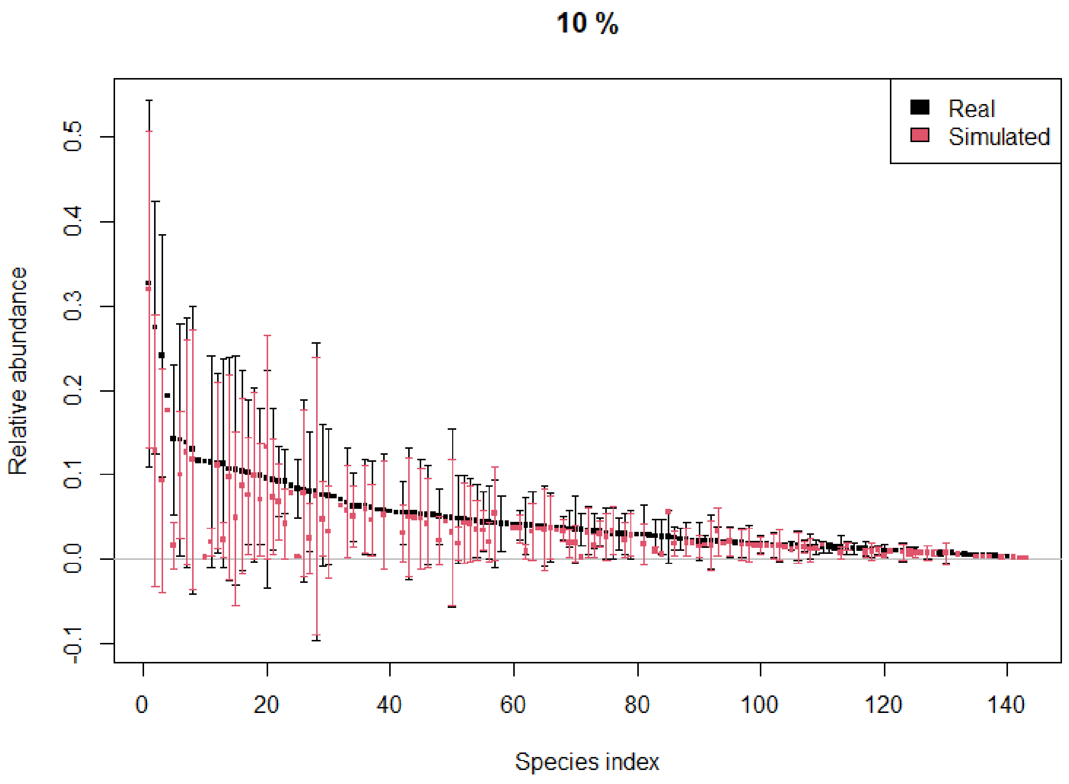

Figure S1. Real *versus* simulated abundances for simulations with 10% new individuals. Dots represent the average abundance and bars represent the standard deviation.

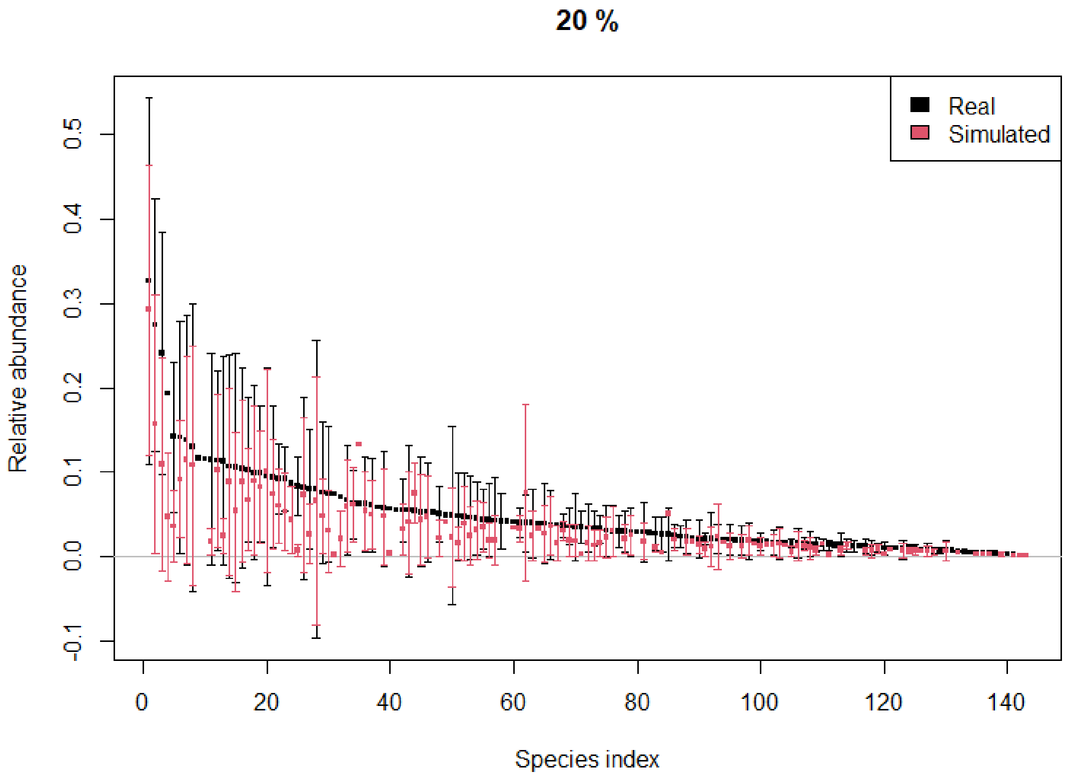

Figure S2. Real *versus* simulated abundances for simulations with 20% new individuals. Dots represent the average abundance and bars represent the standard deviation.

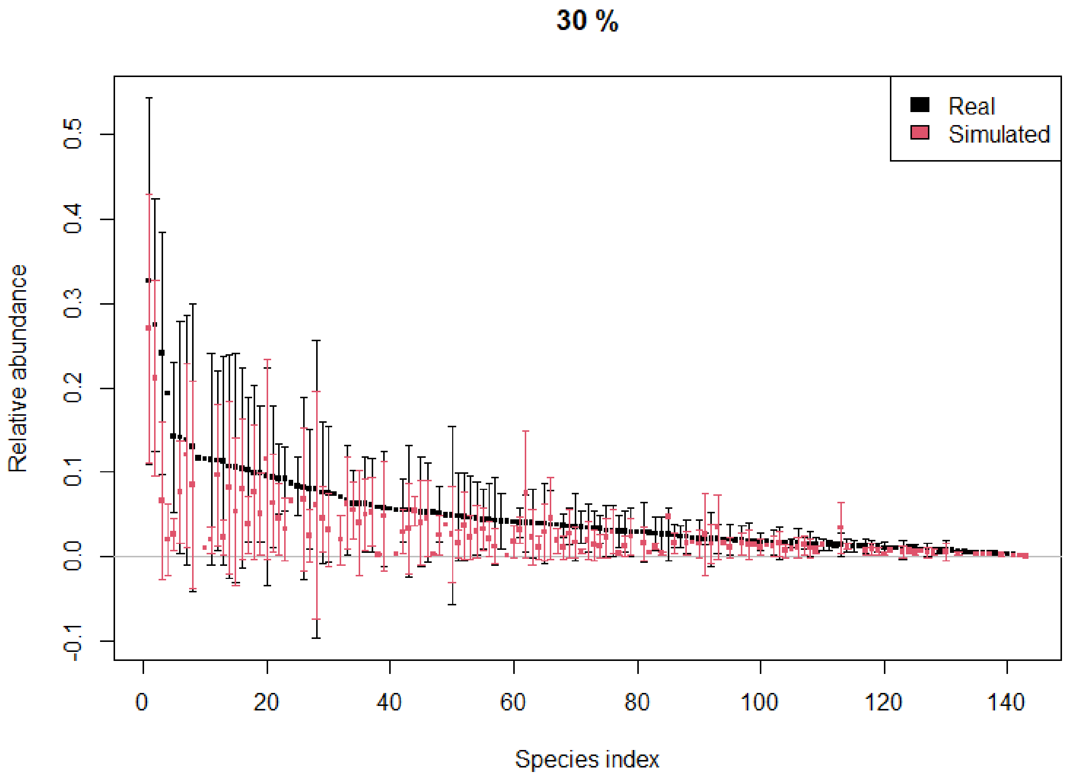

Figure S3. Real *versus* simulated abundances for simulations with 30% new individuals. Dots represent the average abundance and bars represent the standard deviation.

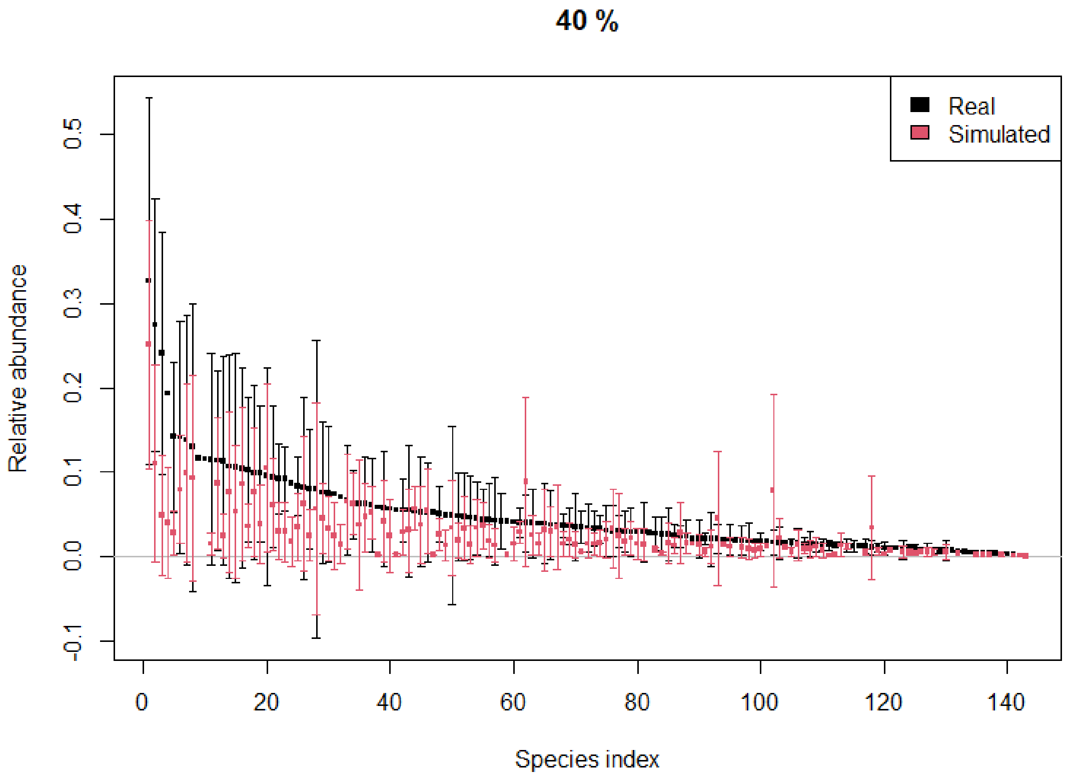

Figure S4. Real *versus* simulated abundances for simulations with 40% new individuals. Dots represent the average abundance and bars represent the standard deviation.

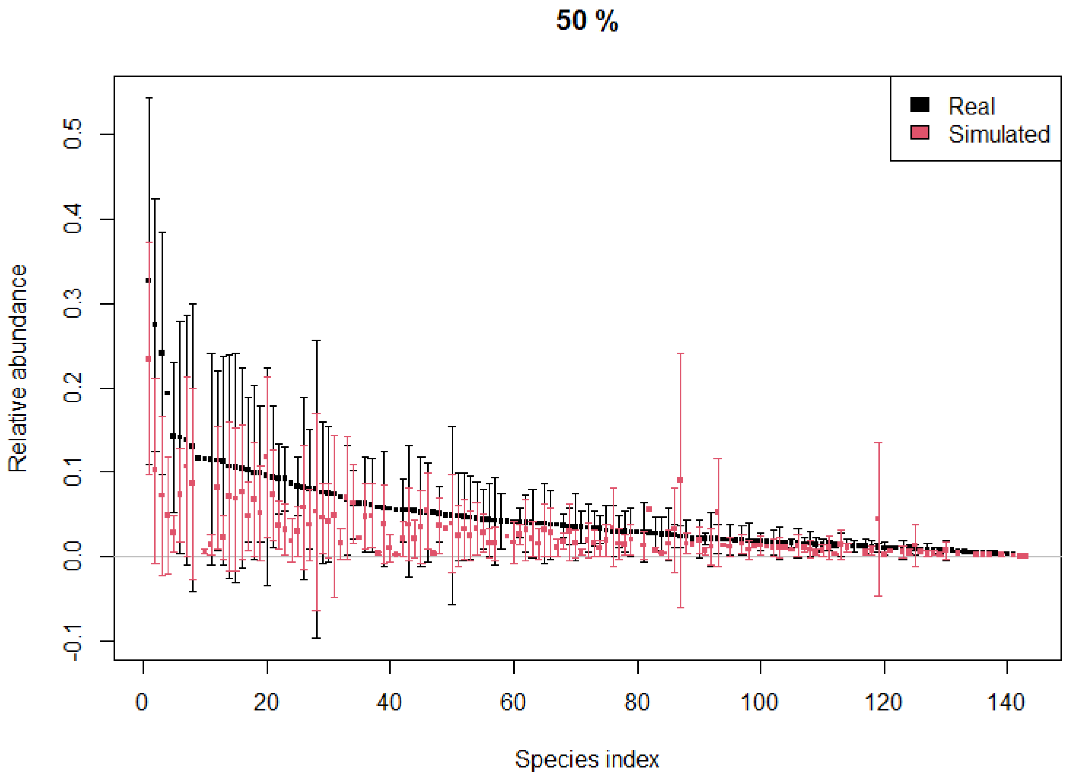

Figure S5. Real *versus* simulated abundances for simulations with 50% new individuals. Dots represent the average abundance and bars represent the standard deviation.

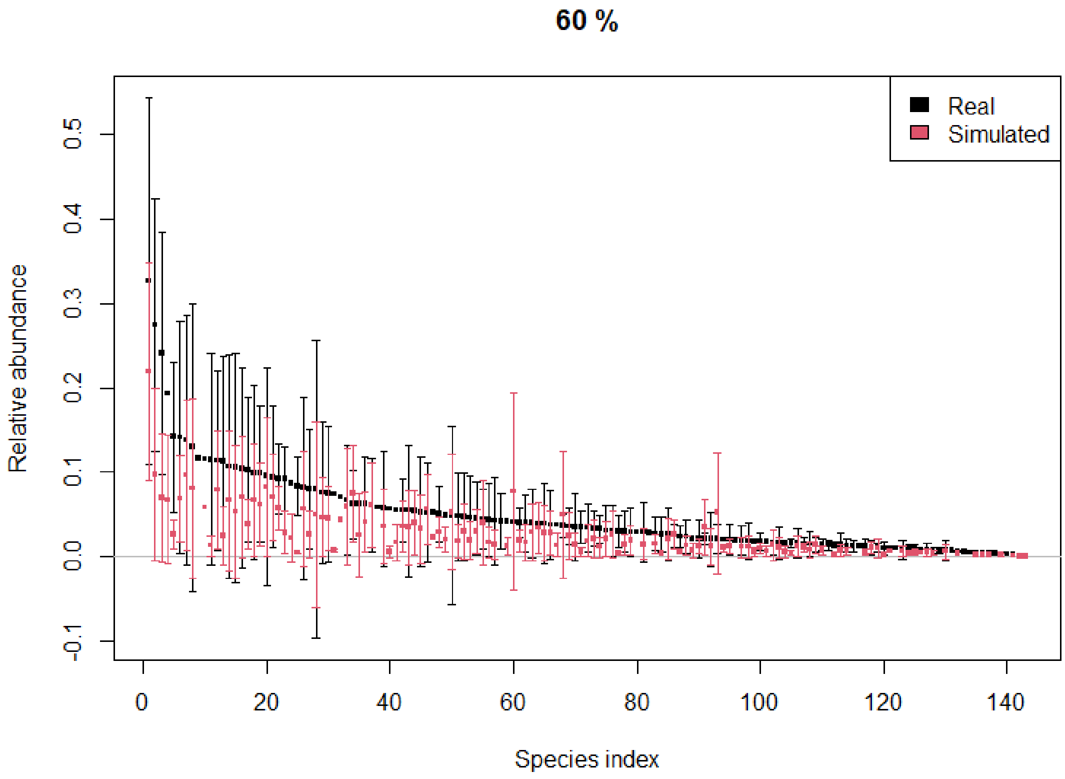

Figure S6. Real *versus* simulated abundances for simulations with 60% new individuals. Dots represent the average abundance and bars represent the standard deviation.

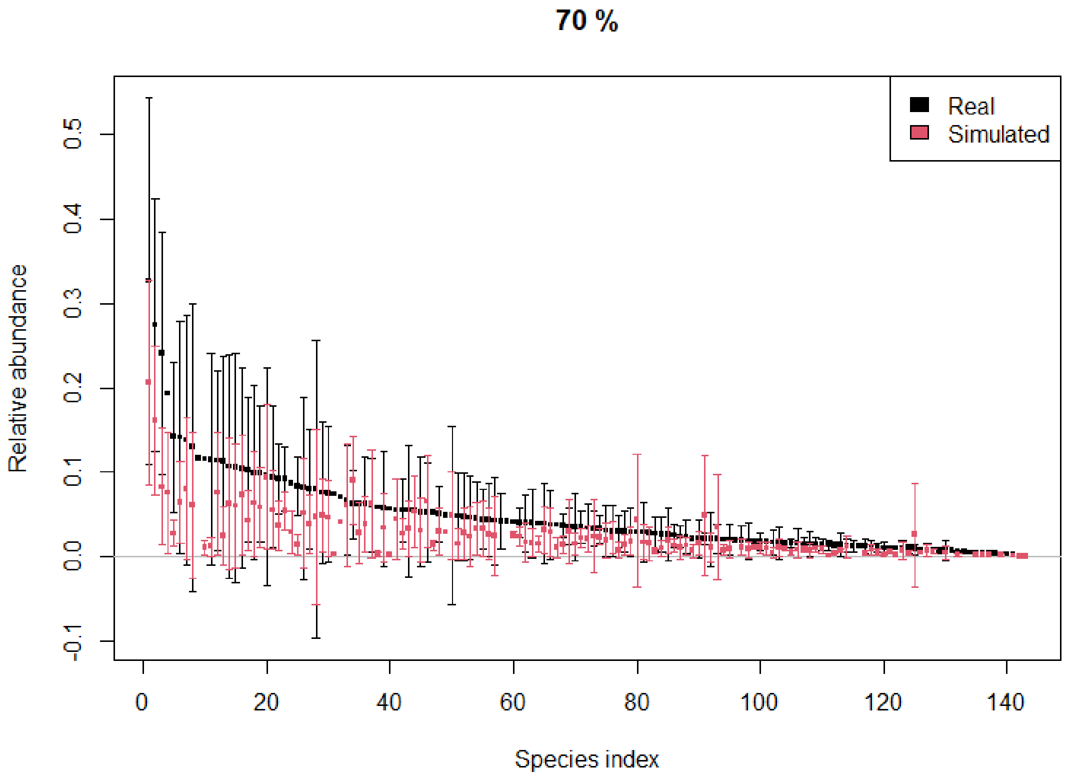

Figure S7. Real *versus* simulated abundances for simulations with 70% new individuals. Dots represent the average abundance and bars represent the standard deviation.

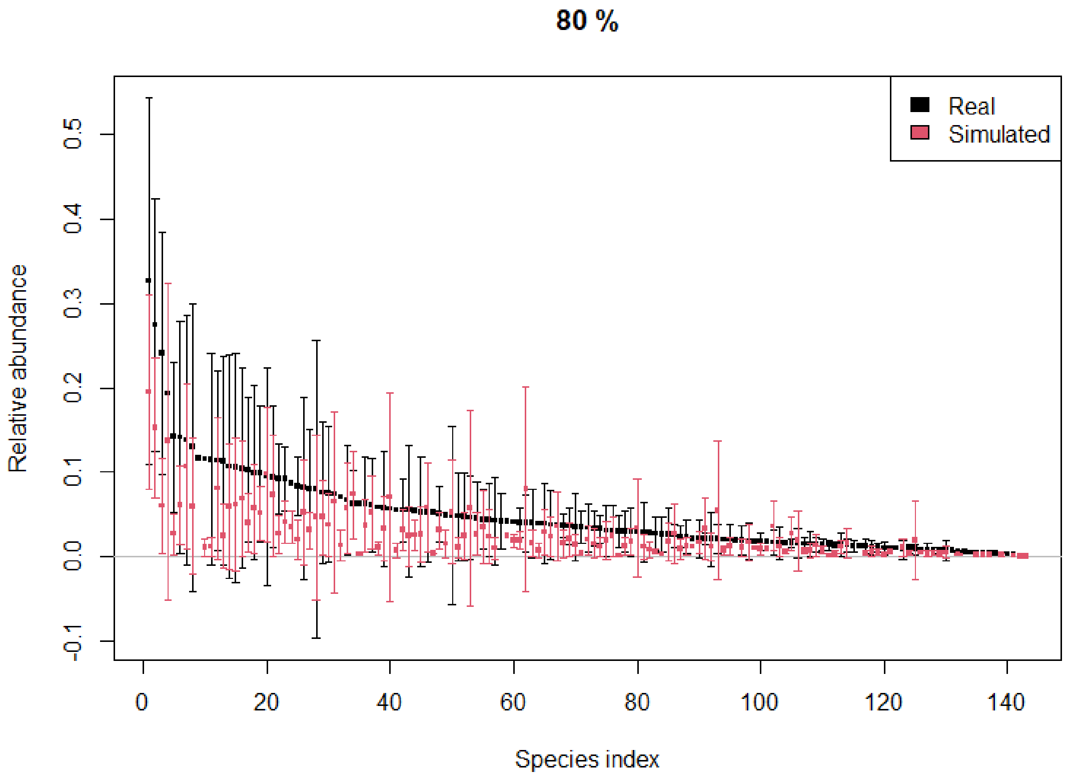

Figure S8. Real *versus* simulated abundances for simulations with 80% new individuals. Dots represent the average abundance and bars represent the standard deviation.

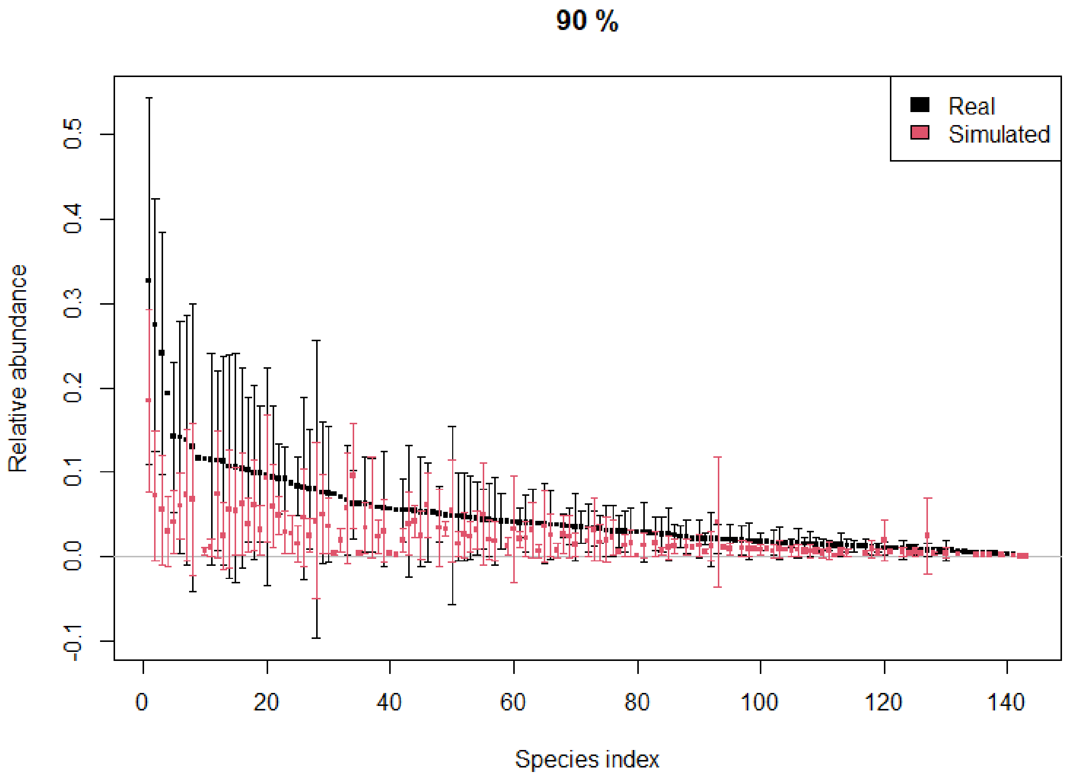

Figure S9. Real *versus* simulated abundances for simulations with 90% new individuals. Dots represent the average abundance and bars represent the standard deviation.

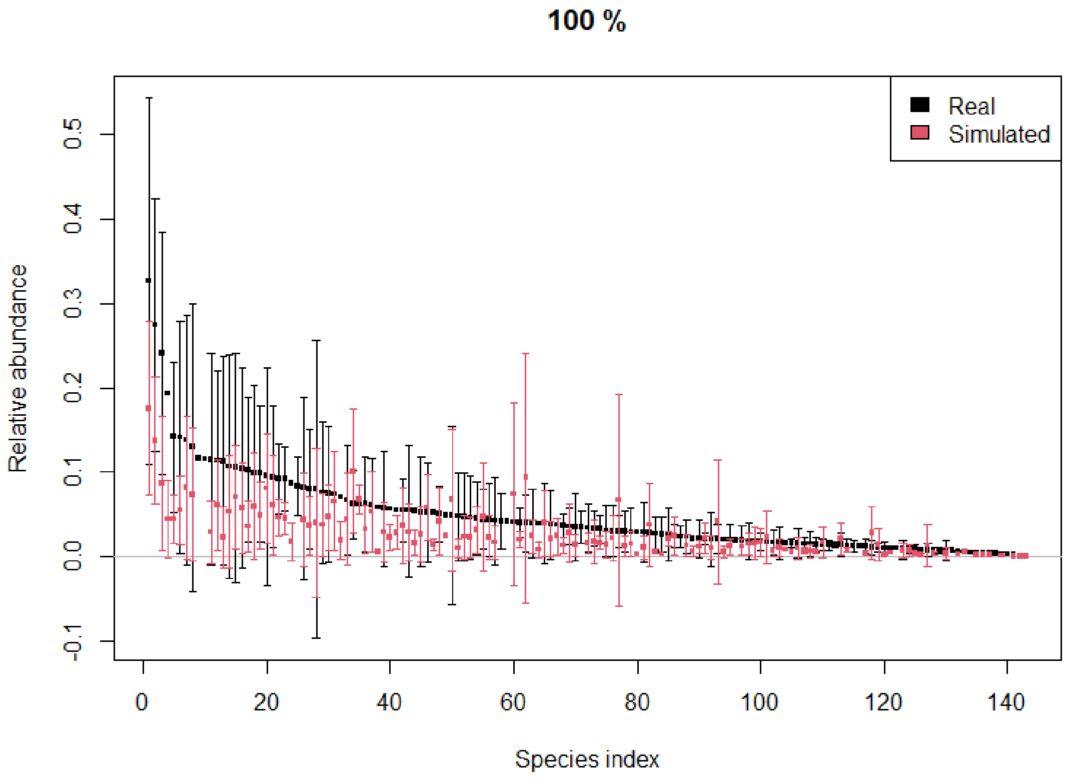

Figure S10. Real *versus* simulated abundances for simulations with 100% new individuals. Dots represent the average abundance and bars represent the standard deviation.

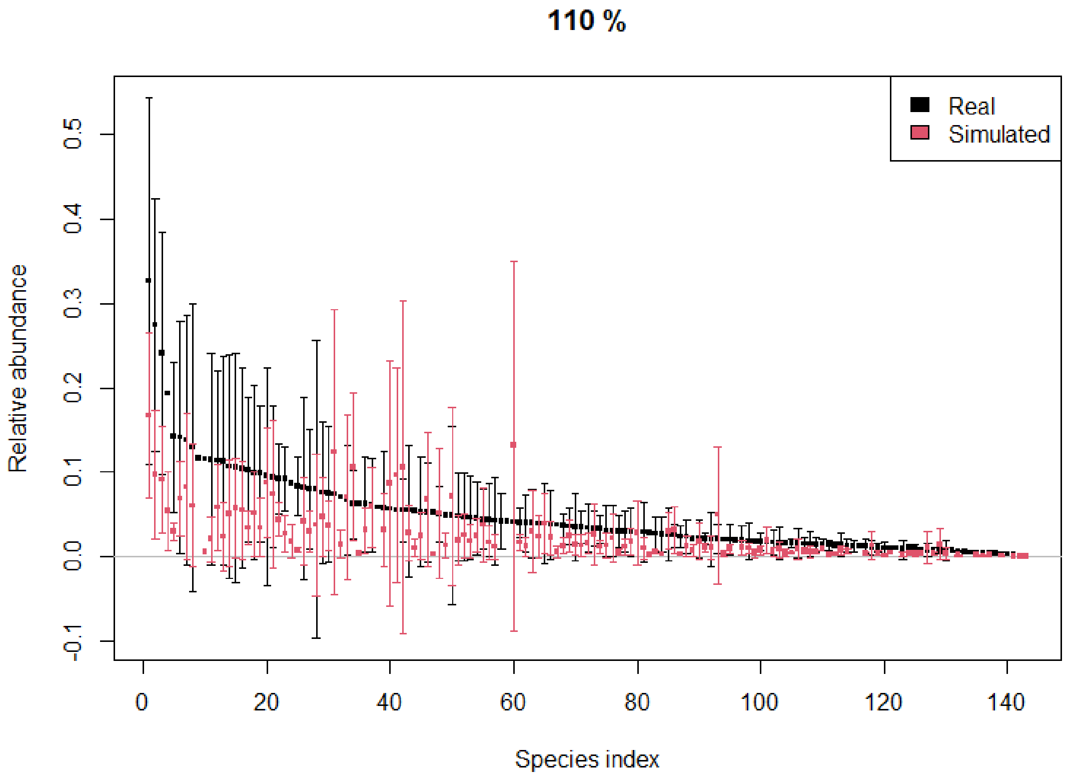

Figure S11. Real *versus* simulated abundances for simulations with 110% new individuals. Dots represent the average abundance and bars represent the standard deviation.

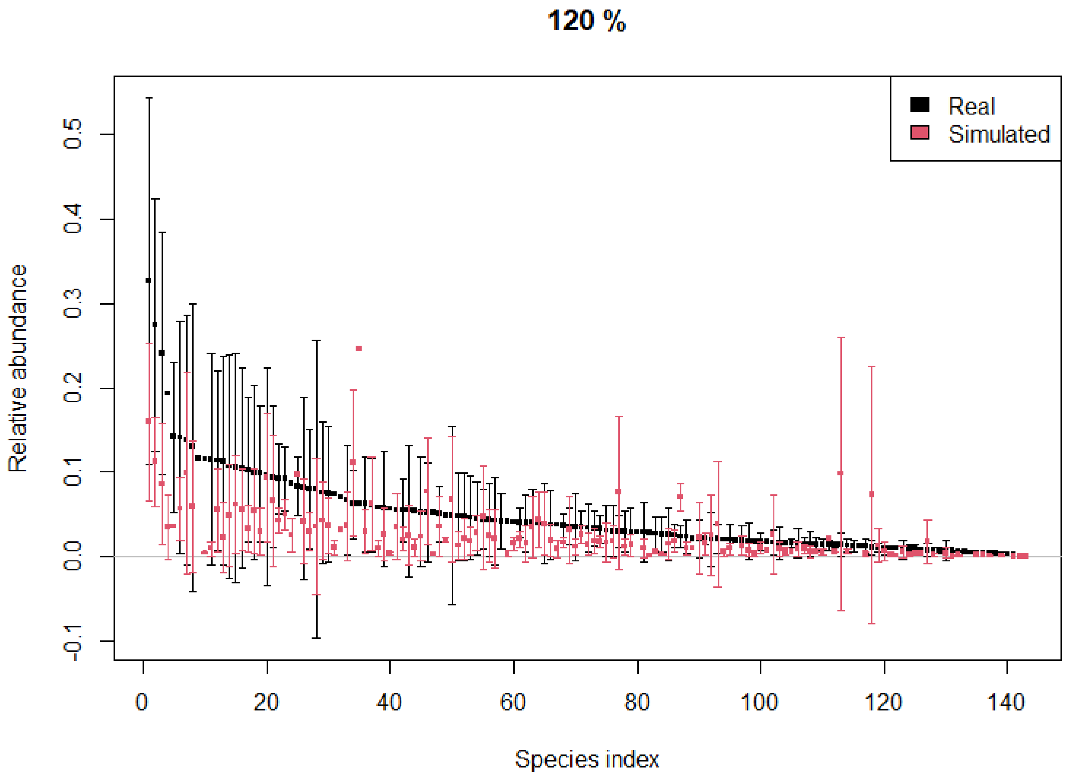

Figure S12. Real *versus* simulated abundances for simulations with 120% new individuals. Dots represent the average abundance and bars represent the standard deviation.

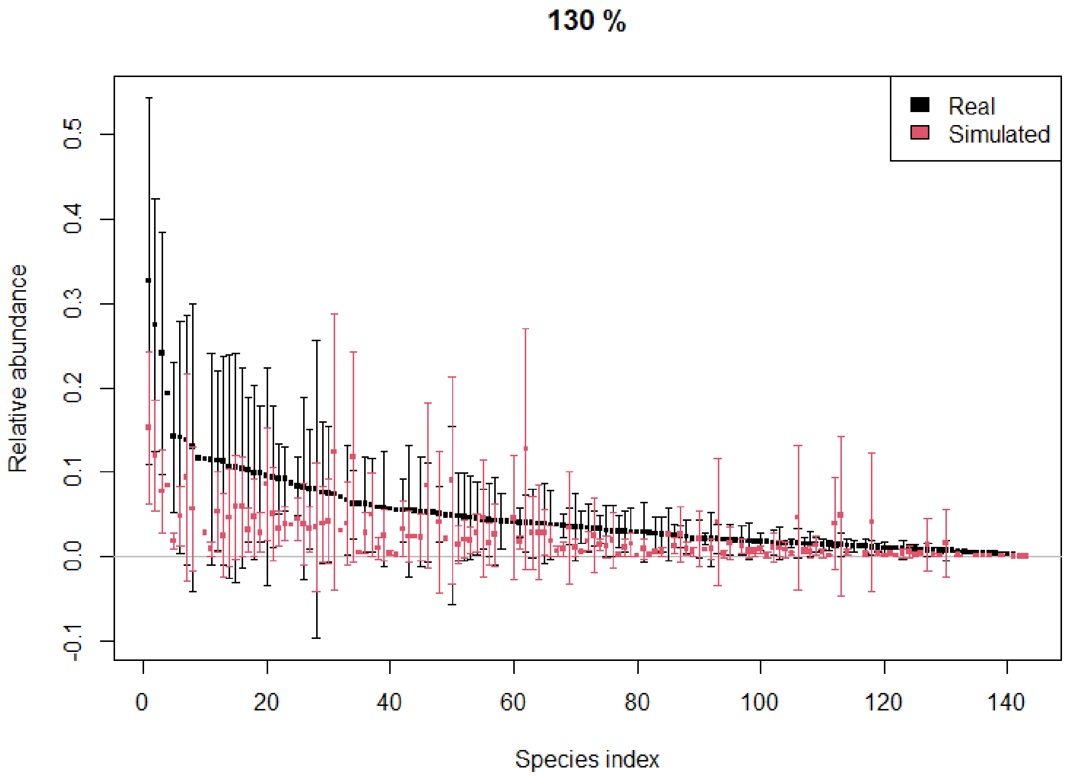

Figure S13. Real *versus* simulated abundances for simulations with 130% new individuals. Dots represent the average abundance and bars represent the standard deviation.

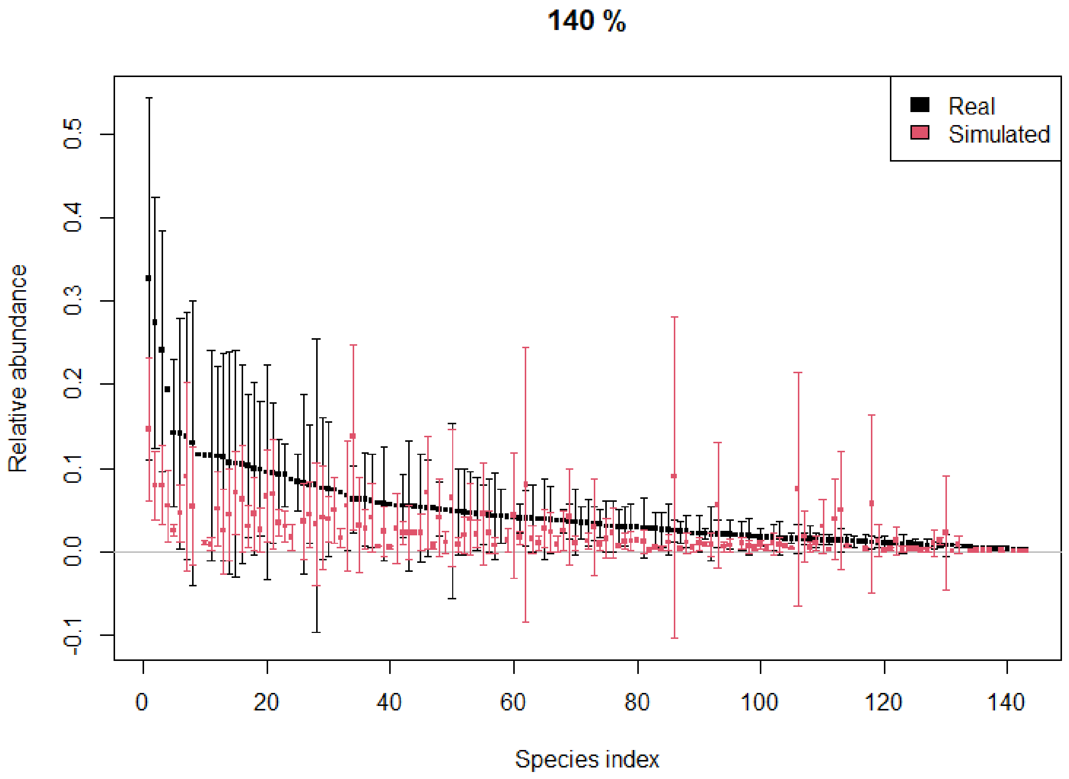

Figure S14. Real *versus* simulated abundances for simulations with 140% new individuals. Dots represent the average abundance and bars represent the standard deviation.

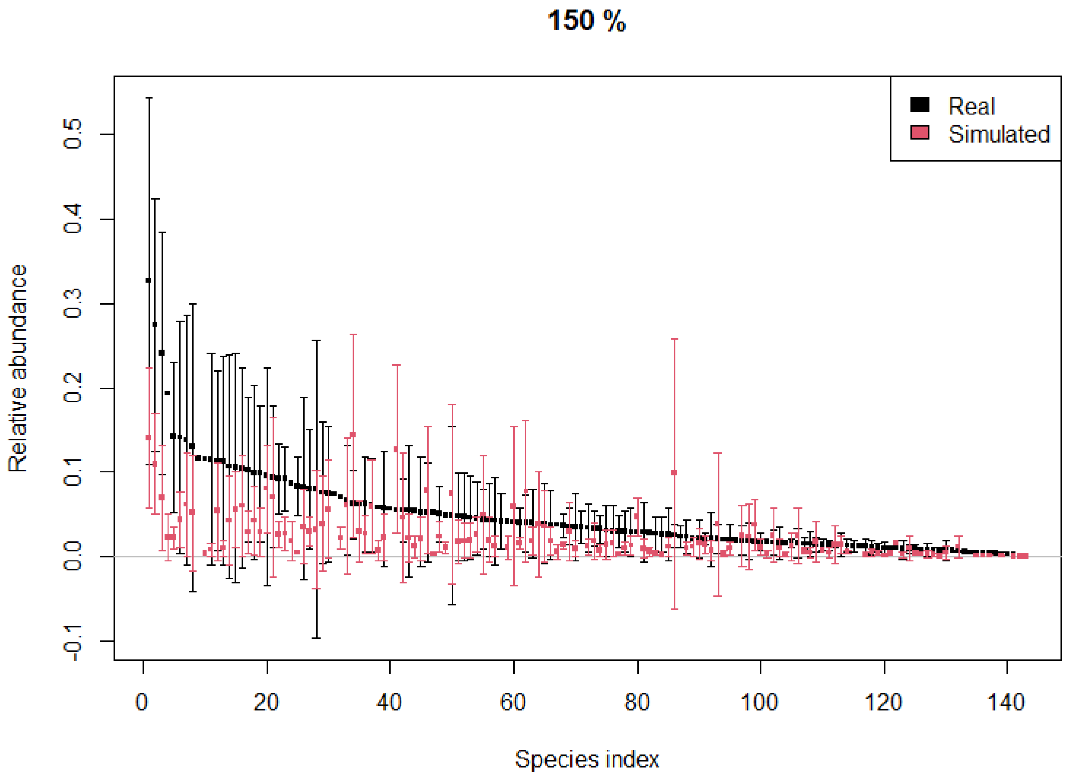

Figure S15. Real *versus* simulated abundances for simulations with 150% new individuals. Dots represent the average abundance and bars represent the standard deviation.

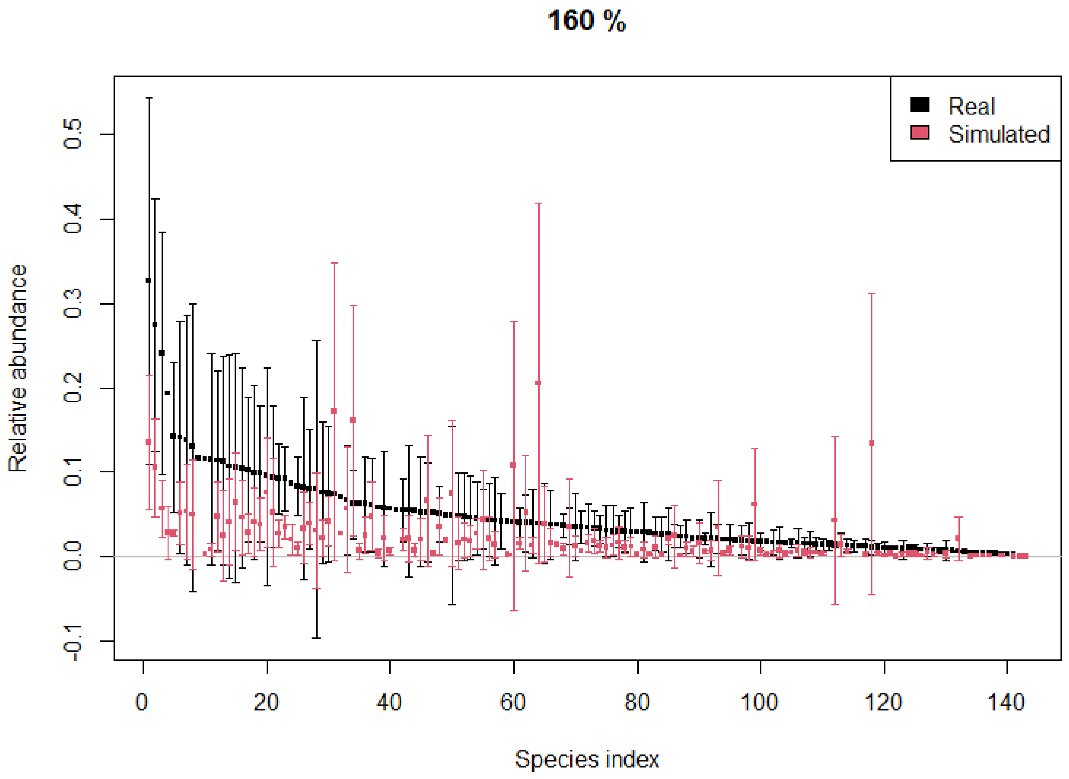

Figure S16. Real *versus* simulated abundances for simulations with 160% new individuals. Dots represent the average abundance and bars represent the standard deviation.

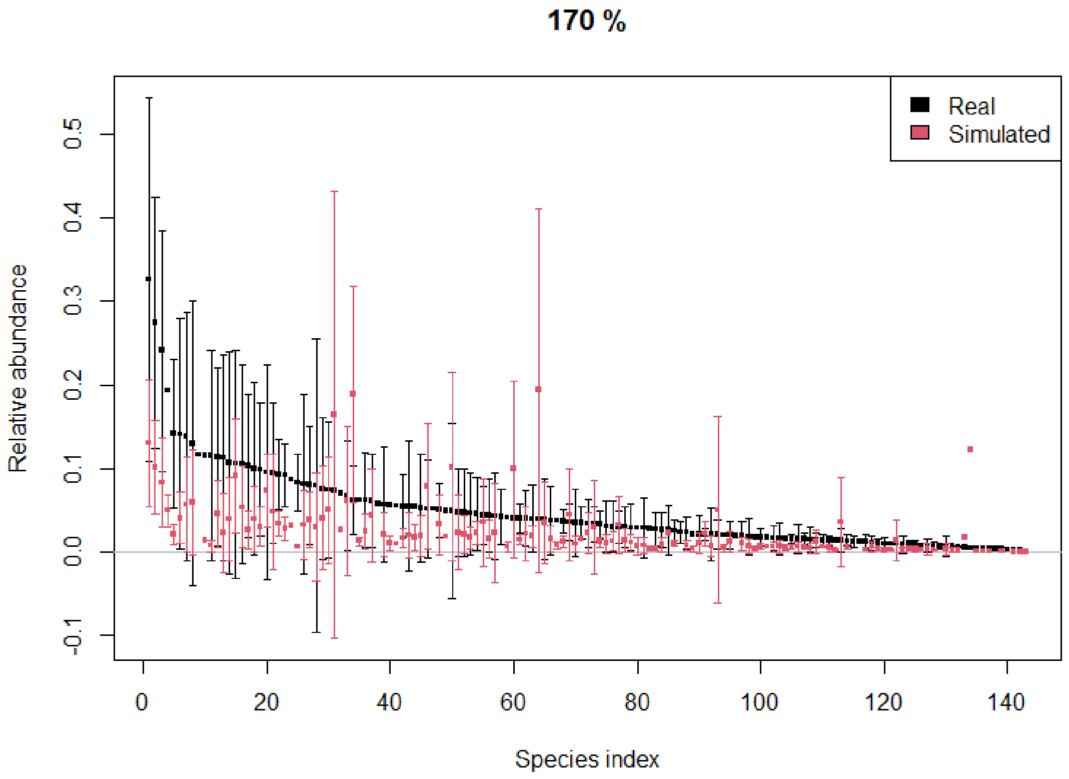

Figure S17. Real *versus* simulated abundances for simulations with 170% new individuals. Dots represent the average abundance and bars represent the standard deviation.

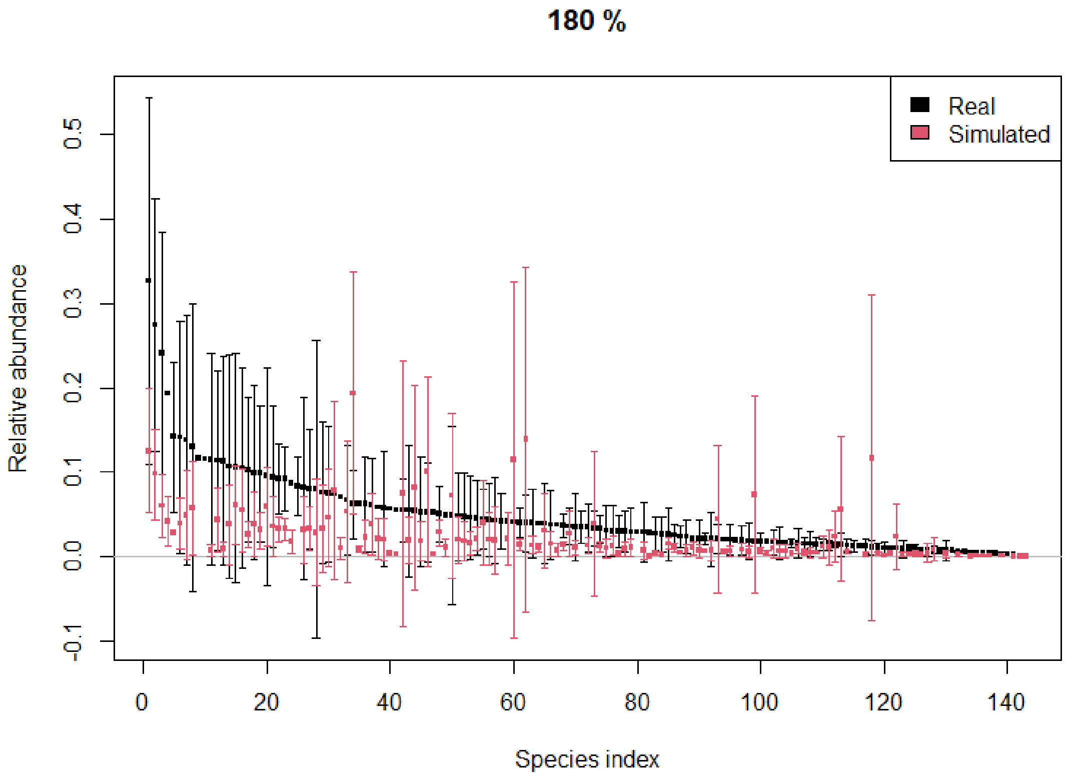

Figure S18. Real *versus* simulated abundances for simulations with 180% new individuals. Dots represent the average abundance and bars represent the standard deviation.

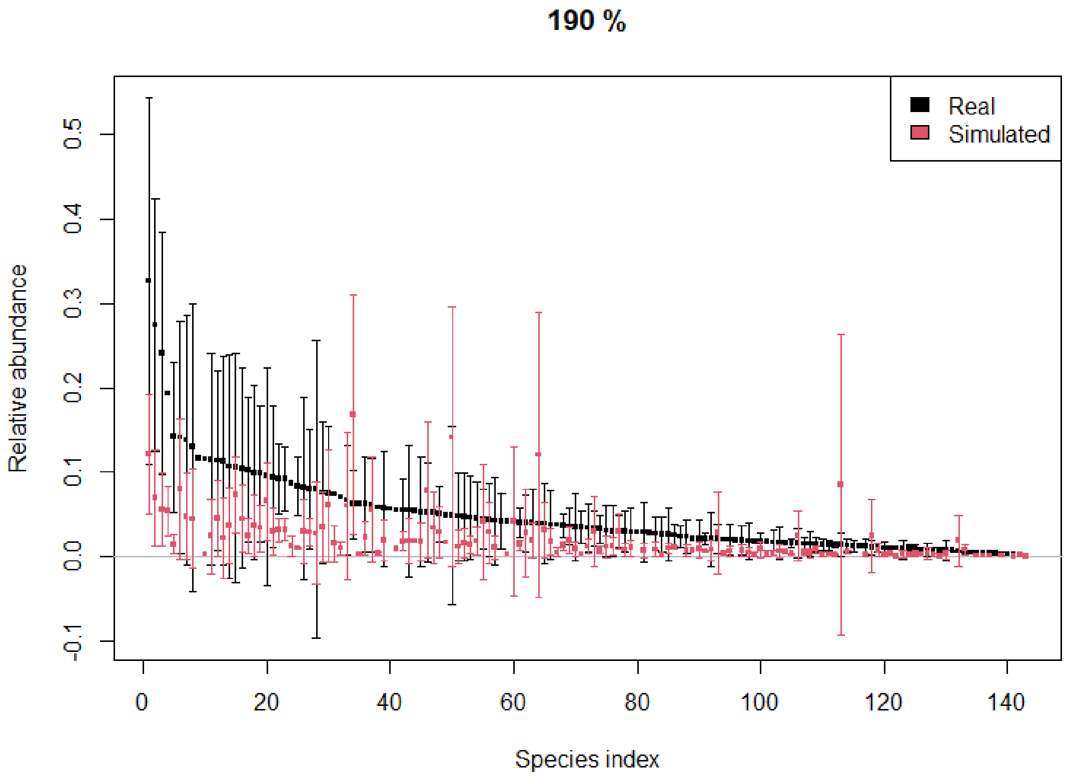

Figure S19. Real *versus* simulated abundances for simulations with 190% new individuals. Dots represent the average abundance and bars represent the standard deviation.

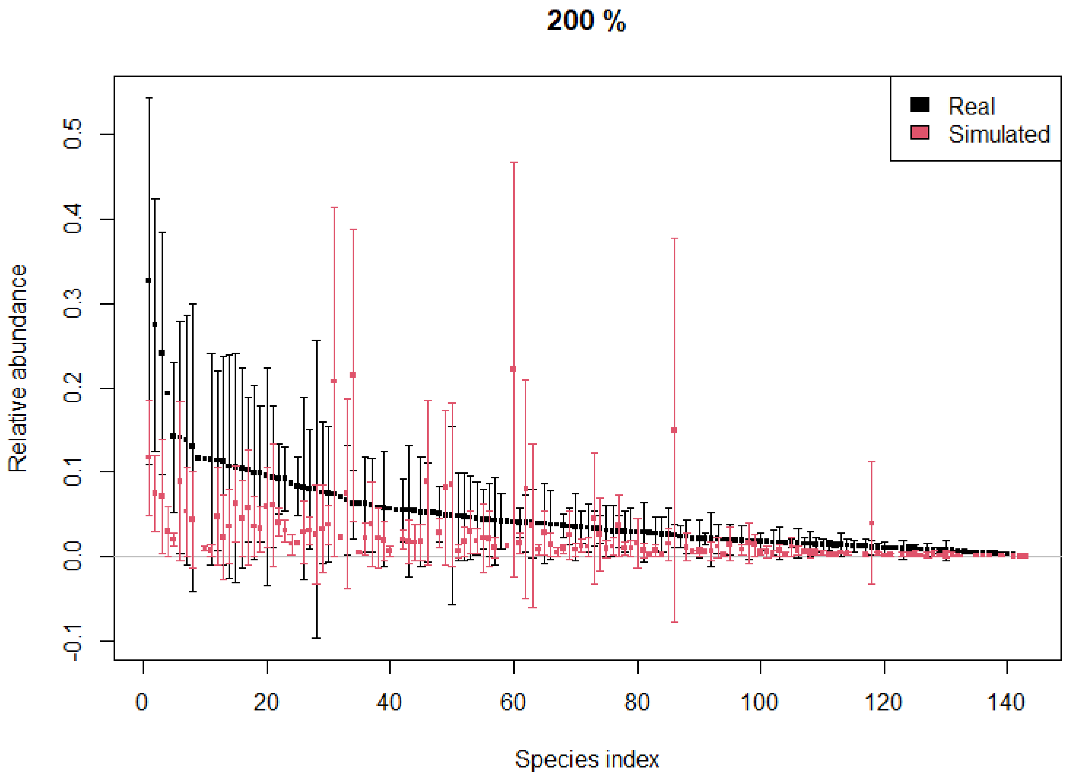

Figure S20. Real *versus* simulated abundances for simulations with 200% new individuals. Dots represent the average abundance and bars represent the standard deviation.

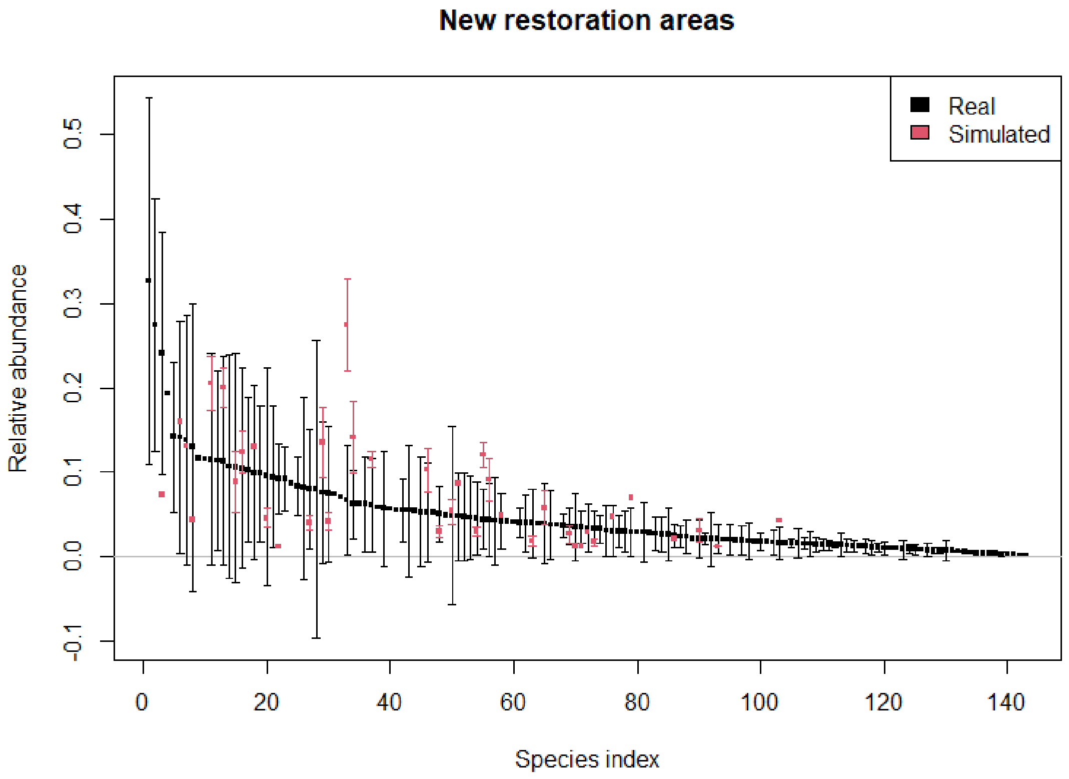

Figure S21. Real *versus* simulated abundances for new restoration areas. Dots represent the average abundance and bars represent the standard deviation.
